# Optimized network inference for immune diseased single cells

**DOI:** 10.3389/fimmu.2025.1597862

**Published:** 2025-07-24

**Authors:** Elena Merino Tejero, Dwain Jude Vaz, Guillermo Barturen, María Rivas-Torrubia, Marta E. Alarcón-Riquelme, Walter Kolch, David Matallanas

**Affiliations:** ^1^ Systems Biology Ireland, School of Medicine, University College Dublin, Dublin, Ireland; ^2^ Pfizer–University of Granada–Junta de Andalucía Centre for Genomics and Oncological Research, Granada, Spain; ^3^ Department of Genetics, Faculty of Science, University of Granada, Granada, Spain; ^4^ Bioinformatics Laboratory, Centro de Investigación Biomédica, Biotechnology Institute, Granada, Spain; ^5^ GENYO, Centre for Genomics and Oncological Research Pfizer, University of Granada, Andalusian Regional Government, Granada, Spain; ^6^ Institute for Environmental Medicine, Karolinska Institutet, Stockholm, Sweden; ^7^ Conway Institute of Biomolecular & Biomedical Research, University College Dublin, Dublin, Ireland

**Keywords:** network inference, Systemic lupus erythematosus (SLE), single-cell, mathematical modeling, gene marker

## Abstract

**Introduction:**

Mathematical models are powerful tools that can be used to advance our understanding of complex diseases. Autoimmune disorders such as systemic lupus erythematosus (SLE) are highly heterogeneous and require high-resolution mechanistic approaches. In this work, we present ONIDsc, a single-cell regulatory network inference model designed to elucidate immune-related disease mechanisms in SLE.

**Methods:**

ONIDsc enhances SINGE’s Generalized Lasso Granger (GLG) causality model used in Single-cell Inference of Networks using Granger ensembles (SINGE) by finding the optimal lambda penalty with cyclical coordinate descent. We benchmarked ONIDsc against existing models and found it consistently outperforms SINGE and other methods when gold standards are generated from chromatin immunoprecipitation sequencing (ChIP-seq) and ChIP-chip experiments. We then applied ONIDsc to three large-scale datasets, one from control patients and the two from SLE patients, to reconstruct networks common to different immune cell types.

**Results:**

ONIDsc identified four gene transcripts: matrix remodelling-associated protein 8 (MXRA8), nicotinamide adenine dinucleotide kinase (NADK), RNA Polymerase III Subunit GL (POLR3GL) and Ultrabithorax Domain Protein 11 (UBXN11) in CD4+ T-lymphocytes, CD8+ Regulatory T-Lymphocytes, CD8+ T-lymphocytes 1 and Low Density Granulocytes that were present in SLE patients but absent in controls.

**Discussion:**

These genes were significantly related to nicotinate metabolism, ribonucleic acid (RNA) transcription, protein phosphorylation and the Rho family GTPase (RND) 1-3 signaling pathways, previously associated with immune regulation. Our results highlight ONIDsc’s potential as a powerful tool for dissecting physiological and pathological processes in immune cells using high-dimensional single-cell data.

## Introduction

Autoimmune diseases are highly heterogeneous conditions, characterized by immune cell dysregulation that plays a central role in pathogenesis (1). Despite their heterogeneity, they share genetic risks, molecular pathway dysregulations, and clinical manifestations (2). Systemic lupus erythematosus (SLE) is one such disease with a prevalence of 43.7 per 100,000 people (3). Longitudinal studies have shown that the interferon (IFN) signature, a set of genes overexpressed upon stimulation of IFN receptors, is associated with clinical phenotypes, disease activity, and accrual damage. These studies often use the SLE Disease Activity Index (SLEDAI) to stratify patient severity (4–6), and have also linked the IFN signature to the activation states of various immune cell types (7–10). Multiple immune cell types in SLE patients exhibit elevated IFN signatures, contributing to tissue inflammation and autoantibody production (11, 12). The role of T lymphocytes is more controversial, likely due to different roles associated with different T lymphocytes subtypes. CD8+ T cells (CD8TCs) and how IFN signatures contribute to autoantibody production and inflammation, but they also exhibit features of exhaustion, which increases infection risk and promotes autoimmunity (13, 14). Furthermore, a reduction in CD8+ regulatory T cell (CD8TREG) subpopulation can result in increased CD4+ T cell (CD4TCs) driving SLE pathogenesis (14). Type I IFN genes activate monocytes (MONOs), B and T lymphocytes (15, 16), while Type II IFNs have paradoxical effects on macrophages and dendritic cells, activating some phenotypes while suppressing others (15). In addition, Type II IFNs promote B cell activation and plasma cell (PC) differentiation in SLE (17). To unravel the complexity of immune-related disease mechanisms, high-resolution mechanistic approaches are required (18). With this perspective, projects like 3TR (taxonomy, treatment, targets and remission) have been designed to better understand the mechanisms of response and non-response to therapy of autoimmune, inflammatory and allergic diseases (19). By investigating shared features across patients and diseases, these efforts may uncover common pathways involved in treatment response and disease progression (20). Typically, bulk ribonucleic acid (RNA) sequencing has been used to measure average gene expression across samples. However, averaging transcriptional information from heterogeneous cell populations can obscure important biological variation. Recent advances in single-cell transcriptomics now allow the study of gene regulatory networks at cellular resolution, offering a more detailed view of immune cell behavior (21, 22).

Mathematical models are powerful tools for generating and testing hypotheses about network structure and function helping to advance our understanding of complex diseases. Machine learning (ML) is making significant strides in SLE research and diagnosis, offering potential advancements in disease understanding, prediction, and treatment. ML algorithms can analyze complex datasets to identify patterns and predict disease activity, flare risks and treatment responses, ultimately aiding in early diagnosis and personalized patient care. While some ML approaches are based on radiologic imaging, others leverage multiomic data sources (23–25). Several network inference algorithms have been developed to reconstruct regulatory networks from bulk, such as Gene Network Inference with Ensemble of trees (GENIE3) (26), and single-cell RNA sequencing data (27–30). Some of these methods, including Single-cell Inference of Networks using Granger ensembles (SINGE) (30), Singular Component Detection and Optimization (SCODE) (31) and Single-Cell Regularized Inference Using Time-Stamped Expression Profiles (SINCERITIES) (32) rely in pseudotime, while others, like Jump Tree (JUMP3) use real-time points (33). The performance of these network inference algorithms has been benchmarked in different publications (30, 34, 35), with GENIE3 consistently ranking among the top-performing methods, as shown by McCalla and colleagues. In these comparisons, SINGE was not evaluated (34). Deshpande and colleagues later showed that SINGE performed favorably in terms of precision and recall to the other four existing methods, including GENIE3, when evaluated using chromatin immunoprecipitation sequencing (ChIP-seq), ChIP-chip, loss of function (LoF), and gain of function (GoF) data (30). SINGE is a kernel-based generalization of the Generalized Lasso Granger (GLG) causality model that infers regulatory networks from pseudotime-ordered single cells. It depends on a large set of hyperparameters. Rather than optimizing them, SINGE aggregates results from a reduced set of hyperparameter combinations to improve robustness. Although this ensemble approach performs better than most individual configurations, it does not achieve the best average precision. The lambda penalty hyperparameter has the greatest impact on performance. Moreover, while ensemble aggregation is suitable for small datasets (<3100 genes), it increases runtime, requires substantial computational resources, and is not scalable to larger datasets. In fact, most benchmarking studies have used relatively small datasets (<2000 and <8000 genes, respectively) (34, 35); whereas real-world patient datasets often contain >20,000 genes.

Finding the optimal set of hyperparameters is essential for generating meaningful networks. Hyperparameters directly influence model performance, affecting metrics such as accuracy, precision, recall, stability, scalability, and runtime (30, 34, 35). Proper hyperparameter tuning also helps balance model complexity, avoid over- or underfitting and allow adapt the model to different datasets. The choice of optimizer depends on the nature of the problem. While simple, differentiable regression problems may be solved using gradient descent (36), more complex, non-differentiable Lasso regression problems require advanced methods such as cyclical coordinate descent. This technique iteratively adjusts one hyperparameter at a time, optimizing the objective function while fixing the rest of the hyperparameters. This approach is computationally efficient and particularly effective for large sparse single-cell datasets, as it breaks down the complex, multivariable optimization problem into a simpler, one-dimensional step-by-step process (37, 38).

**Figure 1:**
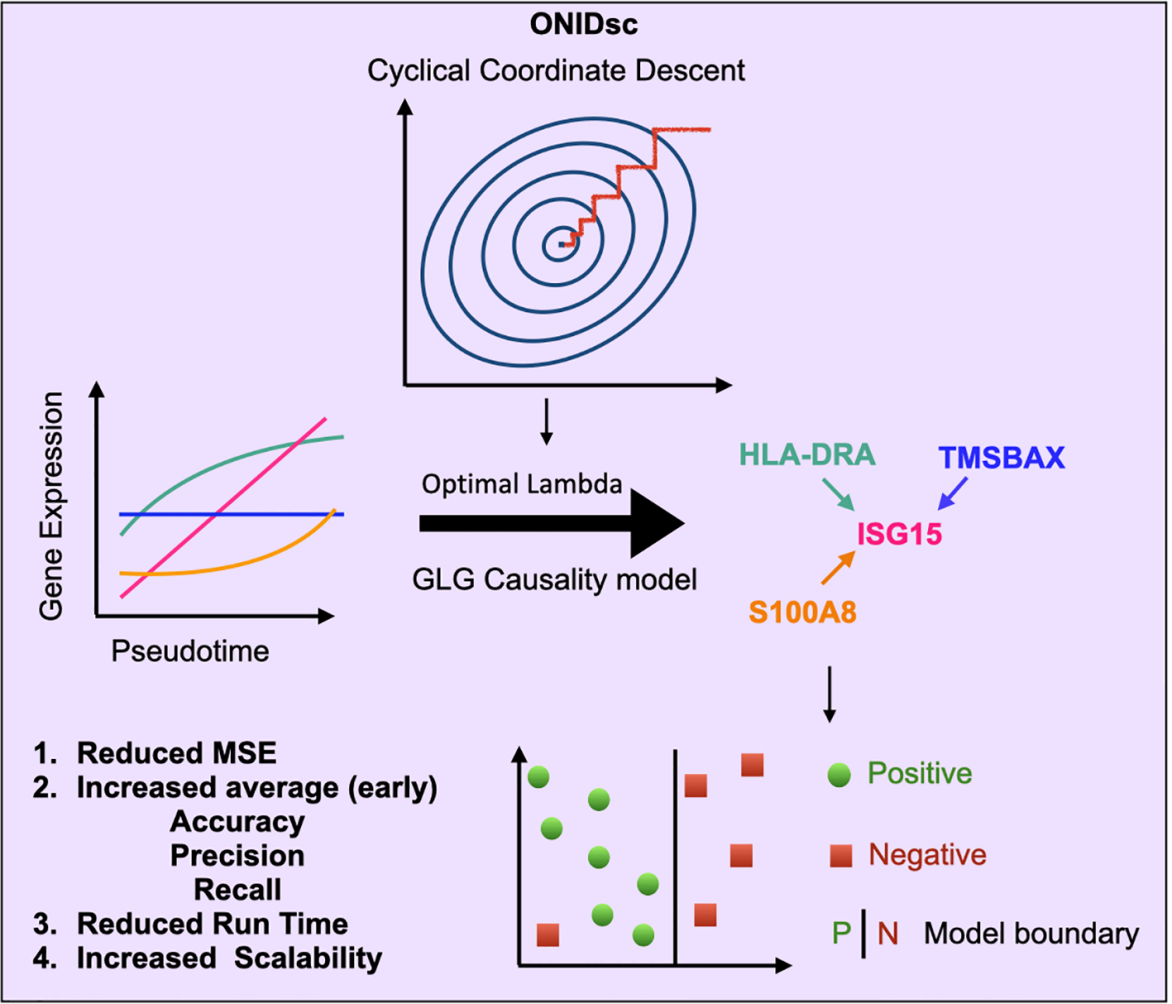
Overview of ONIDsc: a scalable regulatory network inference framework using optimized GLG causality. ONIDsc uses pseudotemporally ordered single-cell gene expression data to infer regulator-target gene networks based on SINGE’s GLG causality test. Unlike SINGE, which uses five fixed lambda hyperparameter values, ONIDsc identifies a single optimal lambda using cyclical coordinate descent. This optimization improves performance by reducing MSE, increasing (early) accuracy, precision, and recall, reducing runtime, and enhancing scalability, making ONIDsc suitable for large patient datasets.

In this study, we introduce ONIDsc, a pseudotemporal-based single-cell network inference algorithm that enhances SINGE’s GLG causality model by finding the optimal lambda penalty with cyclical coordinate descent. We compare ONIDsc network inference to other existing methods and find it consistently outperforms SINGE ensemble. Furthermore, ONIDsc outperformed all methods when gold standards were generated from ChIP-chip and ChIP-seq experiments. We then apply ONIDsc to one control and two SLE patient datasets to reconstruct networks common to different immune cell types. We proposed four genes likely involved in SLE pathogenesis. In summary, we demonstrate that combining advanced optimization with state-of-the-art single-cell network inference enables scalable analysis of large patient datasets and provides new insights into immune-related disease mechanisms (**Figure 1**).

## Materials and methods

### Pre-processing

**Figure 2:**
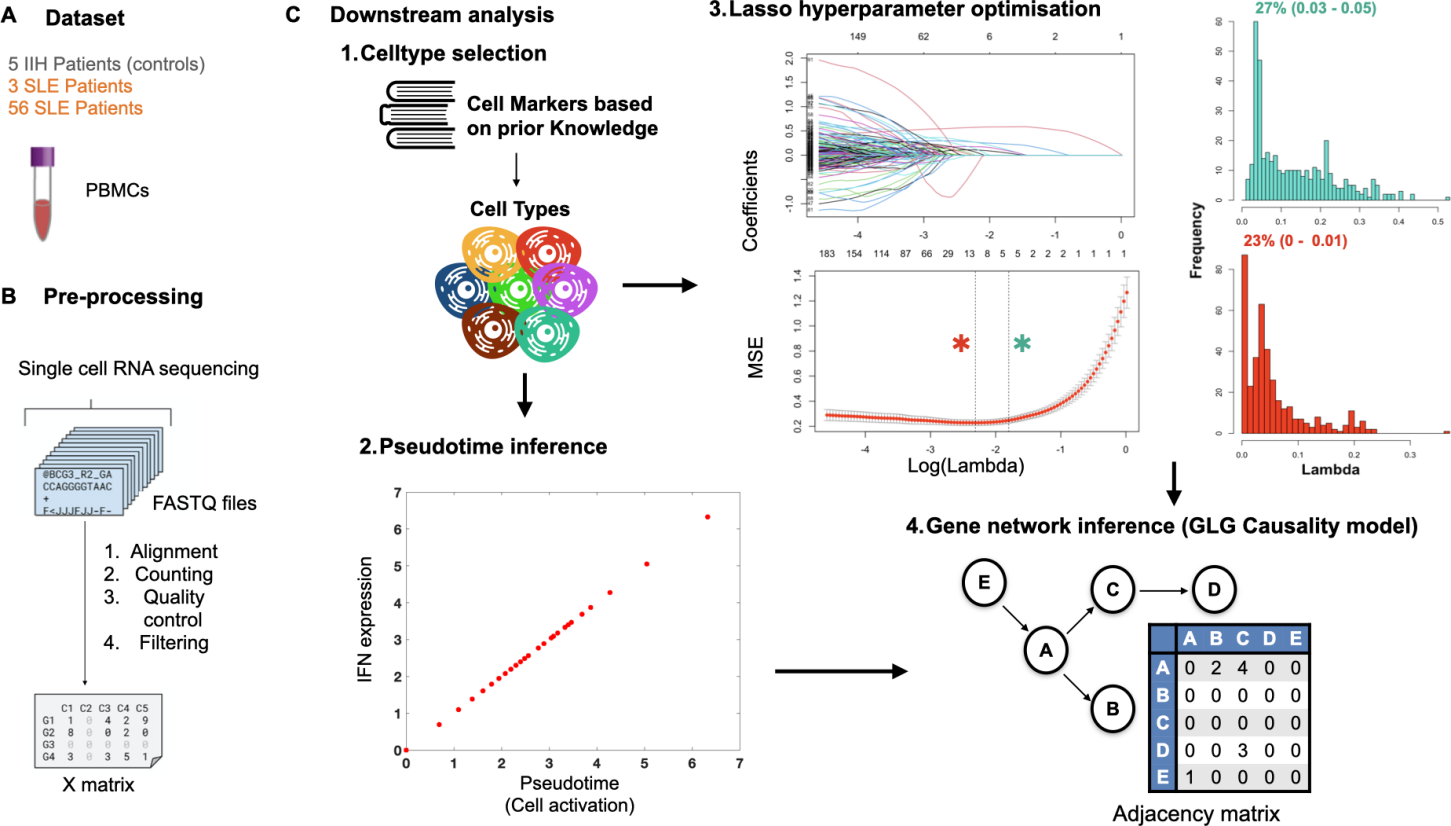
ONIDsc pipeline: from subset selection and preprocessing to network inference. Diagram of the ONIDsc pipeline summarizing **(A)** the subsets analyzed, **(B)** preprocessing steps including normalization and pseudotime ordering, and **(C)** the downstream analysis stages leading to GLG causality-based network inference. The downstream analysis is divided into four main components.

Three scRNA-seq different datasets were analyzed including one with five idiopathic intracranial hypertension (IIH) patients as controls (39), one with three SLE patients (7) and one with fifty-six SLE patients (11) (**Figure 2A**). Pathological characteristics were examined in IIH patients showing oligoclonal bands (proteins indicating inflammation of the central nervous system) were not detected, CSF and blood were unaffected, and there was no other diagnosed disease in the controls (**Supplementary Figure 1** of the referred paper (39)). Thus, they were considered appropriate controls.

All datasets came from peripheral blood mononuclear cells (PBMCs). First, fastq files from sequenced single cells were downloaded from public repertoires (**Figure 2B**). The Schafflick and colleagues (39) dataset was downloaded from the Gene Expression Omnibus (GEO) repository using accession number: GSE141797. The Mistry and colleagues (7) dataset was downloaded from the GEO repository using accession number: GSE142016. The Nehar-Belaid and colleagues (11) dataset was downloaded from the Genotypes and Phenotypes (dbGAP) database using accession number: phs002048.v1.p1. All datasets were pre-processed using the same protocol (For a detailed description, see **Supplementary Material 1**).

### Cell type selection and pseudotime

Single cells from patient datasets were categorized into different immune-related cell types based on prior cell marker analysis (7, 40, 41) (**Supplementary Table 1**; **Figure 2C**). First the different CD8TC subtypes (CD8TC1s, CD8TC2s, CD8TC17s and CD8TREGs) were selected. Intersecting cells between each CD8TC subtype with the other three were removed to avoid redundancy in our analysis. Then CD4TCs, Low Density Granulocytes (LDGs) and MONOs were selected. Intersecting cells between MONOs and CD8TCs, CD4TCs and LDGs were removed from MONO cell types. Finally, B cell subtypes, PCs and memory B cells (MBCs), were selected. Intersecting cells between each of the B cell subtypes and CD8TCs, CD4TCs and LDGs were removed from both B cell subtypes. The level of subtyping was based on the relevance of the given cell population for the disease of interest. After cell typing, single cells within each of the subtypes were ordered along an immune cell activation pseudotemporal process represented by all type I and II IFN-related genes, including IFN alpha, IFN beta, IFN gamma, IFN lambda, IFN epsilon, IFN omega, IFN kappa, Interferon-stimulated Gene 15 (ISG15) and Interferon-stimulated Gene 20 (ISG20) (**Figure 2C**). These genes were used as indicators of the cell activation state due to their established role in activating the studied cell types. The sum of the normalized gene expression of all the IFN-related genes was used as the cell order.

### Lasso optimization

The Lambda hyperparameter was optimized using cyclical coordinate descent. This is an optimization algorithm that operates by sequentially minimizing a multivariable function along one coordinate direction at a time. It begins with an initial guess for the variables and iteratively updates each variable sequentially. In each iteration, all variables except one are fixed, and the function is minimized with respect to the unfixed variable. This process is repeated, typically cycling through the coordinates in a systematic fashion, until the changes in the function value or the variables fall below a predefined threshold, indicating convergence.

We used glmnet package (42) to compute the regularization path for lambda. First, genes with less than four non-zero gene expression values were filtered out. During ten-fold cross-validation, glmnet requires the response variables to have at least five unique values. Genes with constant expression were also filtered out to avoid glmnet failure during the standardization procedure due to their standard deviation being zero. The expression of each gene (response variable) and that of the rest of the genes (predictors) was modelled in the expression matrix. Glmnet function was used to fit a generalized linear model via penalized maximum likelihood (Equation 1) solving the following minimization problem over the entire range of lambda values:


(1) $$min\beta_{0},\beta \frac{1}{N}\sum_{i=1}^{N}\;w_{i}l_{i}\left(y_{i},\beta_{0}+\beta^{T}x_{i}\right)+\lambda \left[\frac{1-\alpha }{2}\|\beta {\|}_{2}^{2}+\alpha \|\beta {\|}_{1}\right]$$

Where *l_i_* (*y_i_,n_i_*) was the negative log-likelihood contribution for observation *i*. Alpha was a tuning parameter used to define the lasso problem by setting it to one. Lambda controlled the severity of the penalty.

The above-mentioned minimization problem was solved using a Gaussian family (Equation 2). Supposing there were observations χ*_i_* ∈ℝ*^p^* and responses *y_i_* ∈ℝ,i=l, ….N. The objective function for the Gaussian family was:


(2) $$min(\beta_{0},\beta )\in {\text{ℝ}}^{p+1}\frac{1}{2N}\sum_{i=1}^{N}{\;(y_{i}-\beta_{0}-x_{i}^{T}\beta )}^{2}+\lambda \left[\frac{1-\alpha }{2}\|{\|}_{2}^{2}+\alpha \|\beta {\|}_{1}\right]$$

Cyclical coordinate descent was then applied to solve the problem (Equation 3). Supposing current estimates ${\tilde{\beta }}_{0}$ and ${\tilde{\beta }}_{l}$ ∀ℓ∈1, …,*p*. By computing the gradient at *βj*= ${\tilde{\beta }}_{j}$ and using simple calculus, the problem was updated to:


(3) $${\tilde{\beta }}_{j}\leftarrow \frac{S\left(\frac{1}{N}\sum_{i=1}^{N}\;x_{ij}\left(y_{i}-\left({\tilde{\beta }}_{0}+\sum_{l\neq }^{j}\;x_{il}{\tilde{\beta }}_{l}\right)\right),\lambda \alpha \right)}{1+\lambda \left(1-\alpha \right)}$$

where *S*(*z, y*) was the soft-thresholding operator with value sign (*z*)(|*z*|−*y*)_+_.

Cross-validation is a crucial component of the Lasso regression fitting process. We used cv.glmnet function to apply k-fold cross-validation in order to find an optimal minimum and maximum lambda for each gene (**Figure 2C**). We used the default number of folds (nfolds = 10), adequate for large datasets. This step involved dividing the data into training and validation sets multiple times to ensure that the chosen lambda minimized the mean squared error (MSE) in a robust and generalizable manner. The distribution of minimum and maximum lambdas showed a severe left-skewed distribution. Thus, the most frequent minimum and maximum for all genes were computed. We analyzed the distribution of minimum and maximum lambdas and the MSE per gene of different lambda values within the optimal range for several datasets.

### GLG network inference

Ordered single-cell RNA-seq data were taken as input and analyzed using multiple GLG instances with different hyperparameters. Each hyperparameter set resulted in an adjacency matrix (gene by gene matrix) and each element in the adjacency matrix represented the weight of the relation between a gene pair (**Figure 2C**). Genes could be regulators and/or targets. Non-zero elements were then ranked and aggregated using a modified borda count, with an optional subsampling stage increasing the effective ensemble size. The main hyperparameters included the lambda, the number of replicas, the time resolution, the extent of the lagged time series, the kernel width, the zero handling and the probability of zero removal. Default values specified in SINGE were used for all hyperparameters except for the probability of zero removal, lambda and the number of replicas. We have used a probability of zero removal of 0.2 in all our benchmarking and patient dataset analysis. Thus, for each gene, each zero-valued sample and its corresponding pseudotime were removed with a probability of 0.2. This was done to mitigate the dropout severity of the patient samples. Interestingly, the effect of changing the zero-removal probability was previously studied, showing that the average early precision and average precision were marginally affected. Cyclical coordinate descent was used to find a single lambda hyperparameter value within the optimal range. Eight technical replicas per hyperparameter combination were analyzed to reduce resources consumed, since it was found that from five replicas on, the average early precision plateaued (30).

### Benchmarking

Two small-sized datasets and their corresponding gold standards were used for benchmarking purposes. The first dataset was the embryonic stem cell (ESC) to endoderm differentiation dataset from Hayashi and colleagues (43) with 356 cells and 100 genes. As a gold standard, 652 known interactions were taken from the embryonic stem cell atlas from the pluripotency evidence ESCAPE database (44). To generate the gold standard, 95% of ChIP-chip/ChIP-seq and 5% LoF and GoF interactions were combined. The second dataset was the retinoic acid dataset from Semrau and colleagues (45) with 1886 cells and 626 genes. As a gold standard, 1862 known interactions were taken from the embryonic stem cell atlas from the pluripotency evidence ESCAPE database (44). To generate the gold standard, 52% of ChIP-chip/ChIP-seq and 48% LoF and GoF interactions were combined. To ensure the reliability of the gold standard used for benchmarking, we employed the ESCAPE database, one of the most comprehensive repositories of experimentally validated interactions in mouse embryonic stem cells (ESCs). ESCAPE integrates data from multiple experimental sources, including ChIP-seq, RNAi, and protein-protein interaction studies. Interactions not documented in ESCAPE were assumed to be absent.

**Figure 3:**
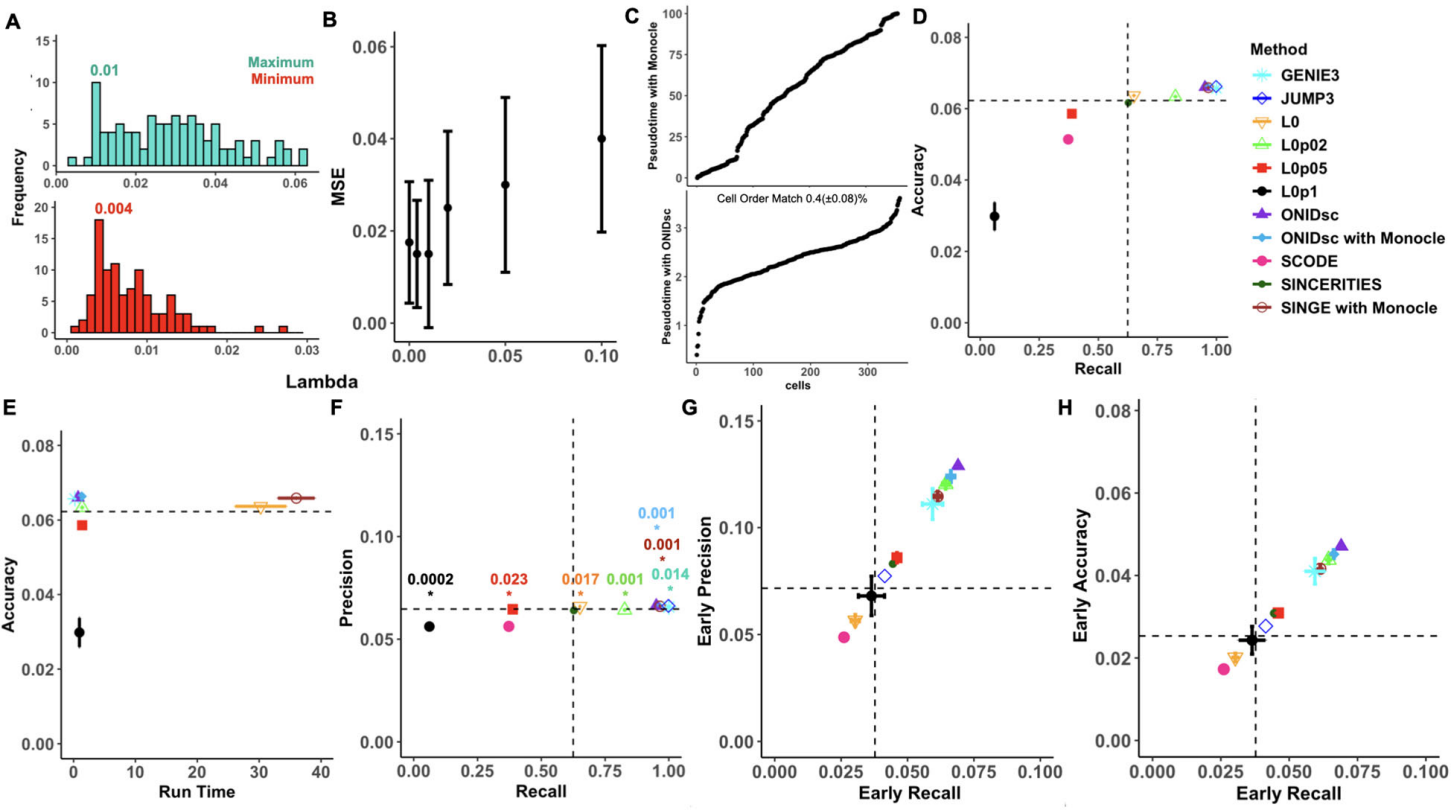
Benchmarking ONIDsc against regulatory network and pseudotime methods on ESC-to-endoderm differentiation data. **(A)** Minimum (red) and maximum (green) optimal lambdas. **(B)** MSE distribution over lambdas proposed by SINGE and ONIDsc. **(C)** Comparison of pseudotime inference between ONIDsc and Monocle. **(D)** Accuracy over recall, **(E)** accuracy over run time, **(F)** precision over recall, **(G)** early precision over early recall and **(H)** early accuracy over early recall. Six regulatory network inference algorithms were tested: GENIE3 (light blue), JUMP3 (dark blue), ONIDsc (purple), ONIDsc’s network inference combined with Monocle’s pseudotime (blue), SCODE (fuchsia), SINCERITIES (dark green) and SINGE combined with Monocle’s pseudotime (brown). SINGE was also run with each of the lambda hyperparameter values individually: L0 (orange), L0p02 (light green), L0p05 (red), and L0p1 (black). Four replicas were run for GENIE3, ONIDsc and SINGE. The standard deviation is represented with bars. T-tests comparing the average precision of ONIDsc, with GENIE3 and SINGE were performed. Statistically significant differences (P value< 0.05) were represented with an asterisk and the p value is indicated above. Both asterisks and p-value are colored by the method ONIDsc was compared with. Methods are also represented with different shapes as indicated in the figure legend.

We benchmarked ONIDsc against five other existing regulatory network inference methods: SINGE (30), SINCERITIES (32), SCODE (31), JUMP3 (33), and GENIE3 (26). Default settings were used to run all methods. SINGE’s ensemble outputs was run four times using the same default hyperparameters for both benchmarking datasets: 10 replicas, [0,0.01,0.02,0.05,0.1] lambdas, [ (3,5) (5,9) (9,5) (5,15); (15,5)] time resolution and number of lag combinations, [0.5; 1; 2; 4] kernel widths, 0 zero handling and the 0.2 probability of zero removal. Additionally, we run SINGE with one of the benchmarking datasets using default values except for the lambda parameter, which was set to [0], [0.02], [0.05], [0.1] individually to assess the performance of each single lambda value (**Figure 3**). SINGE was always used in combination with Monocle for pseudotime inference (28). ONIDsc was also run four times on both benchmarking datasets using the following hyperparameters: 10 replicas, [ (3,5) (5,9) (9,5) (5,15); (15,5)] time resolution and number of lag combinations, [0.5; 1; 2; 4] kernel widths, 0 zero handling and the 0.2 probability of zero removal. The embryonic stem cell (ESC) to endoderm differentiation dataset was run using [0.01] optimal lambda (**Figure 3**) while the retinoic acid dataset was run using [0.1] optimal lambda (**Supplementary Figure 1**). The Lambda parameter value used to run ONIDsc was within the optimal range. GENIE3, originally developed for bulk transcriptomics, was applied without cell ordering or pseudotime. JUMP3 used cell ordering information, while the remaining methods relied on pseudotime. SCODE was run with four degrees of freedom for the ESC to endoderm dataset and twenty degrees of freedom for the retinoic acid dataset to account for the larger gene network size.

We also benchmarked ONIDsc against another pseudotime inference method, Monocle (28), which was combined with SINGE’s and ONIDsc’s network inference to order single cells along a pseudotime (46). ONIDsc pseudotemporal concept was applied to both benchmarking datasets by ordering the cells based on the sum of the log expression of the genes known to drive the differentiation process in each of them. For the embryonic stem cell (ESC) to endoderm differentiation dataset, the following gene markers were used: *Undifferentiated Embryonic Cell Transcription Factor 1 (UTF1)*, *Myelocytomatosis Oncogene (MYC)*, *Krüppel-like Factor 4 (KLF4)*, *Sex Determining Region Y-Box 2 (SOX2)*, and *Octamer-Binding Transcription Factor 4 (OCT4)*, based on previous studies demonstrating their involvement in ESC differentiation (47). For the retinoic acid differentiation dataset, the following pluripotency-associated markers were used: *Zinc Finger Protein 42 (ZFP42)*, *POU Class 5 Homeobox 1 (POU5F1)*, *KLF4*, *Developmental Pluripotency Associated 5A (DPPA5A)*, *Estrogen-Related Receptor Beta (ESRRB)*, *SOX2*, *DPPA3*, *Fibroblast Growth Factor 4 (FGF4)*, *Krüppel-like Factor 2 (KLF2)*, and *Transcription Factor CP2-Like 1 (TFCP2L1)*, based on prior findings linking them to pluripotency (45).

To evaluate the quality of regulatory network predictions, we used standard performance metrics including accuracy, precision, and recall. Accuracy measures the overall correctness of the model’s classifications. Precision assesses the proportion of true positives among predicted positives, penalizing false positives, while recall evaluates the model’s ability to identify all true positives, penalizing false negatives. To assess the top-ranked regulator–gene interactions—often the most biologically relevant in experimental settings—we also computed early accuracy, precision, and recall. These early metrics were calculated using partial recall thresholds of 0.1 for the ESC to endoderm differentiation dataset and 0.3 for the retinoic acid dataset. The method runtime was evaluated for GENIE3, SINGE, and ONIDsc. To determine whether the evaluated methods performed better than random, we generated control networks by assigning random interactions between genes from each dataset. Method variability was assessed using four independent runs for GENIE3, SINGE, ONIDsc, and the random control.

### Software and resources

ONIDsc was implemented using a variety of softwares. Cell type selection and pseudotime inference algorithms were built in Matlab (48) (version R2022a). The Lasso optimization algorithm was built in R (49) (version 4.3.2) using various libraries: stringr (version 1.5.1), BiocManager (version 1.30.22), e1071 (version 1.7-14), ggplot2 (version 3.5.0), dplyr (version 1.1.4), glmnet (version 4.1-8), Matrix (version 1.6-5), ggtext (version 0.1.2), readr (version 2.1.5), pheatmap (version 1.0.12). Gene regulatory network inference algorithm SINGE (version 0.5.1) was provided by on Matlab-based Docker containers. Docker images were converted to singularity (50) (version 2.4) to be run on two high-performance computing (HPC) clusters. GENIE3 was run in parallel using R packages: GENIE3 (version 1.26.0), doParallel (version 1.0.17) and doRNG (version 1.8.6). Network visualization was done using Cytoscape (51) (version 3.10.2) and pathway enrichment analysis (PEA) was performed using Reactome (52) (version 88), gene ontology (GO; version 3.18.0) (53) and R (49) (version 4.3.2). Simulations were done on Sonic HPC cluster, for use by the UCD research community, and the Kay cluster of Ireland’s national HPC cluster. Each dataset was run using LongQ and large partitions on Kay and Sonic clusters. Serial batches were resourced with one node, eight central processing units (CPUs) and 150 gigabites (GBs) of random-access memory (RAM). Runs were split over different serial batches. Jobs were parallelized between two and thirty-six times, depending on the size of the dataset which determined the run duration. ONIDsc source code is publicly available on GitHub (https://github.com/ElenaMerinoTejero/ONIDsc-master.git). For a more detailed analysis of the resources used to analyze the Mistry and colleagues (7) dataset with SINGE and ONIDsc algorithms, see **Supplementary Files 1** and **2**. For a detailed core and runtime analysis of the Nehar-Belaid and colleagues dataset (11) run with ONIDsc see **Supplementary File 3**.

## Results

### ONIDsc overview and benchmarking

We developed a network inference algorithm, ONIDsc, in order to enhance SINGE’s GLG causality model which was previously described by Deshpande and colleagues (30). ONIDsc is based on SINGE and uses pseudotemporally ordered single-cell gene expression data to predict a regulator-target network that underlies a biological process. The GLG causality model uses a statistical hypothesis, Granger Causality, which tests the predictive causality between a regulator and its target within a time series. Instead of real-time series, the relative position of the cells within a trajectory or pseudotime representing a given biological process was inferred. We avoided using common pseudotime methods that use all genes from the dataset, in an unsupervised manner, to derive the cell order. Instead, we first performed a Log X+1 normalization, to reduce the data skewness and then ordered single cells within each cell type category based on the sum of the normalized expression of IFN-related genes (**Figure 2C**). IFN-related genes represent the cell activation process. These genes are expressed during immune cell activation (54) and have also been found to correlate with disease severity (4). This approach ensured that the cell order specifically reflected the biological process under investigation. Granger Causality combined with lasso regression allows for adjusting model complexity by performing variable selection and regularization to avoid under- or overfitting. The degree to which variables are selected depends on the lambda hyperparameter. ONIDsc introduces the use of cyclical coordinate descent to identify the optimal lambda, which allows the model to adjust the degree of variable selection to each dataset, thereby enhancing performance.

To assess the ability of ONIDsc to improve other existing state-of-the-art methods, we performed benchmarking using two different datasets. We benchmarked the full ONIDsc method, based on a prior marker-based pseudotemporal ordering concept combined with optimal GLG causality network inference against five regulatory network inference methods and one pseudotime ordering method. The five regulatory network inference methods included SINGE (30), based on GLG causality using five different lambdas, SINCERITIES (32), based on ridge regression Granger causality, SCODE (31), based on ordinary differential equations, JUMP3 (33), based on decision trees and GENIE3 (26), a tree-based method. The pseudotime ordering method was Monocle (28), an established method based on an unsupervised reversed graph embedding approach paired with clustering techniques.

First, we applied ONIDsc to the ESC to endoderm differentiation dataset with 95% of the interactions coming from ChIP-chip and ChIP-seq, and 5% from LoF and GoF data. We found the optimal minimum and maximum lambda per gene using a cyclical coordinate descent algorithm. The most frequent minimum and maximum lambdas were 0.004 and 0.01, respectively (**Figure 3A**). Lambdas outside the optimal range resulted in increased MSE (**Figure 3B**). ONIDsc and Monocle pseudotime methods were compared (**Figure 3C**) and combined with ONIDsc network inference method to assess the contribution of each part of ONIDsc to the overall method performance. We assessed performance in terms of accuracy, precision, recall, and runtime, focusing on early or top-ranked regulator-gene interactions (**Figures 3D–H**). The full ONIDsc method, combining its own pseudotime inference and network inference, was the best-performing method in five out of seven performance metrics, namely precision, accuracy, early precision, early recall, and early accuracy. For the remaining metrics, in terms of recall, the ONIDsc full method was outperformed by GENIE3, JUMP3, SINGE ensemble, and ONIDsc network inference when combined with Monocle’s pseudotemporal order. SINGE’s ensemble outputs and ONIDsc combined with Monocle were tied in recall. In terms of runtime, the ONIDsc full method was the second fastest after GENIE3, while significantly improving upon SINGE’s ensemble outputs. Overall, ONIDsc full method was the best-performing method for this dataset. SCODE performed worse than random across all metrics. When examining the performance of SINGE run with suboptimal lambdas individually, we found ONIDsc full method to outperform all individual lambdas across all metrics. Lambda 0.1 performed similarly to or worse than SINGE’s ensemble and random across all metrics. Lambda 0 also performed worse than SINGE’s ensemble and random in the three early metrics and had a runtime nearly as long as SINGE’s ensemble. The fact that certain suboptimal lambdas showed increased MSE along with drastically reduced performance may explain why ONIDsc outperformed both SINGE’s ensemble and SINGE with suboptimal lambdas in six out of seven performance metrics: precision, accuracy, runtime, early precision, early recall, and early accuracy.

We then applied ONIDsc to the retinoic acid dataset, with 52% of the interactions coming from ChIP-chip and ChIP-seq and 48% from LoF and GoF data. Most frequent minimum and maximum lambdas were 0.03 and 0.2 (**Supplementary Figure 1A**). Lambdas outside the optimal range resulted in increased MSE (**Supplementary Figure 1B**). Performance assessment revealed JUMP3 as the best method in five out of seven performance metrics: precision, accuracy, early precision, early recall, and early accuracy. In terms of recall, JUMP3 and SINCERITIES were tied as the best methods. GENIE3 was the fastest method (**Supplementary Figures 1C–G**). The full ONIDsc method ranked fifth in six out of seven performance metrics, namely precision, recall, accuracy, early precision, early recall, and early accuracy. Interestingly, we observed a slight performance improvement when combining ONIDsc network inference with Monocle’s pseudotemporal ordering. This combination ranked fourth in six out of seven performance metrics. ONIDsc outperformed SINGE’s ensemble outputs across all metrics. SCODE again performed worse than random.

Finally, we explored the cellular topology of both benchmarking datasets using t-distributed stochastic neighbor embedding (t-SNE) dimensionality reduction (**Supplementary Figures 2A, B**). This allowed us to better understand the underlying patterns and relationships in each dataset. The ESC to endoderm differentiation dataset displayed a more linear or single-path topology (**Supplementary Figure 2A**), while the retinoic acid dataset exhibited a tree-like, bifurcated, or multi-path topology (**Supplementary Figure 2B**). This difference in topology may explain why ONIDsc’s pseudotime inference outperformed Monocle in the ESC dataset but underperformed in the retinoic acid dataset.

Altogether, these results show that cyclical coordinate descent can enhance the GLG causality test by identifying the optimal degree of variable selection, resulting in reduced MSE and improved (early) precision, accuracy, and recall. They also suggest that ONIDsc may be better suited for identifying interactions from ChIP-chip and ChIP-seq experiments, which are primarily direct (causal), than from gene expression data (LoF and GoF), which may include indirect (correlational) interactions. Finally, ONIDsc’s pseudotime inference method appears more effective for single-cell datasets with linear or single-path topologies.

### Case studies

#### Five IIH patient dataset: network inference of controls using ONIDsc

As the next step, we applied ONIDsc to the dataset from Schafflick and colleagues (39). This analysis aimed to identify baseline networks inferred by ONIDsc in a normally functioning immune system. Seven different immune cell types were analyzed for each of the five controls, resulting in a total of thirty-five cell-type subsets. We found between 3 and 406 cells per cell type, and between 269 and 20,984 genes (**Supplementary Figure 3**). We investigated the shared networks across different control subsets. Networks consisted of a ranked list of scored regulator-target gene relations. We performed clustering of the different cell types based on all relations (**Supplementary Figure 4A**). We found one shared relation in CD8TC2s (cluster 7) and 21 shared relations in MBCs (cluster 4) common to two out of the five controls. This finding illustrates the high heterogeneity of regulatory relationships in the immune system, likely due to the stochastic nature of immune processes at various levels (55). Clusters 1, 3, and 4 contained the most common relations across two of the seven cell types studied (**Supplementary Figure 4B**). We analyzed all nodes from the most common relations using pathway enrichment analysis (PEA) (**Supplementary Figure 4C**). Five out of the thirty-five genes were significantly associated with pathways including fatty acid and vesicle synthesis, cell cycle, mitochondrial RNA processing, and tumor necrosis factor receptor superfamily (TNFRSF) binding involved in apoptosis and inflammation. Importantly, to discard that the use of IHH patients as controls could affect our study, we use a previous large-scale study profiling single cells from healthy human blood across 166 individuals aged 25–85 identified the top 100 differentially expressed genes between major PBMC subpopulations (56). Many members of the TNFRSF, mitochondrially encoded nicotinamide adenine dinucleotide hydrogen ubiquinone oxidoreductase core subunit (MT-ND), and Huntingtin Interacting Protein 1 (HIP1) genes were among them (56). Other studies have also confirmed the role of Palmitoyl-protein Thioesterase 1 (PPT1) (57), regulator of chromosome condensation 1 (RCC1) (58), and TNFRSF9 (59) in a healthy immune system. Additionally, HIP1 receptor expression has been reported to be high in normal PBMCs and lymphoid tissues (60). Altogether, these findings indirectly validate the genes identified as significant in the control population using the ONIDsc method.

We also analyzed all genes present in one of the five controls for CD4TCs and CD8TCs (**Supplementary Figure 5A**). The union of these genes was subjected to PEA (**Supplementary Figure 5B**). We found evidence of CD4TC activation, including pathways related to mitochondrial activity, Vascular Endothelial Growth Factor (VEGF) signaling, SUMOylating in DNA damage repair, and tumor protein p53 (TP53) acetylation, all of which are characteristic of antiviral responses (61). We also found evidence of CD8TC activation, such as Rho family GTPase (RND)1–3 cell cycle pathways. RND proteins are involved in the regulation of the actin cytoskeleton, cell adhesion, and cell cycle, and may also mediate innate defense against viral and bacterial infections (62). Finally, we did not observe any signs of CD4TC or CD8TC stress, nor signs of CD8TC exhaustion, as expected in a healthy and functional immune system.

#### Three SLE patient dataset: ONIDsc vs SINGE resource comparison:

**Figure 4:**
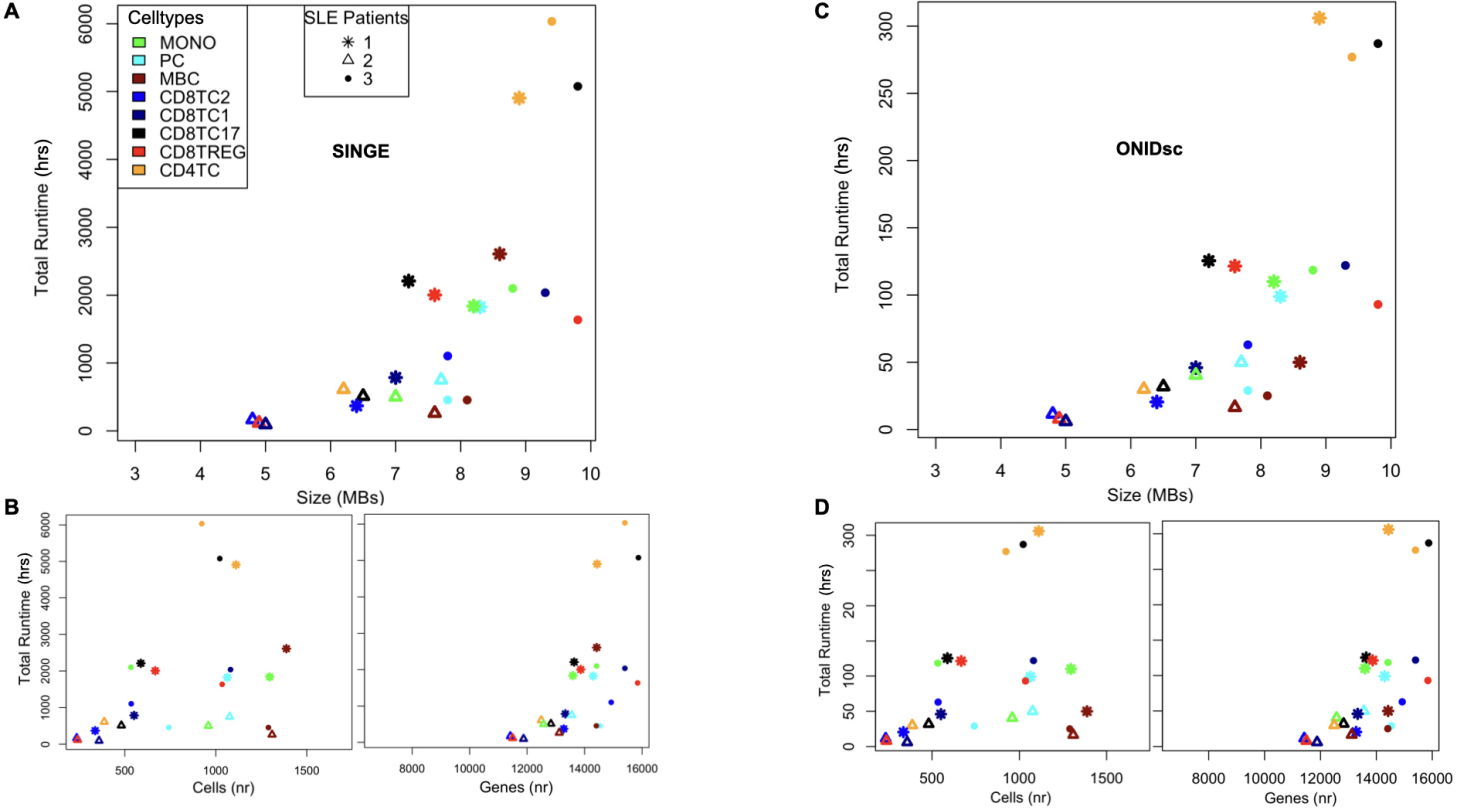
ONIDsc vs. SINGE: runtime and scalability comparison on the SLE patient dataset. **(A)** Total run time in hours (hrs) as a function of subset size in megabytes (MBs) for each subset analyzed from the Mistry and colleagues dataset. **(B)** Total run time as a function of the number (nr) of cells (Left) and genes (Right) for each cell type and patient. **(C)** Total run time as a function of subset size for each cell type and patient. **(D)** Total run time as a function of cells (Left) and genes (Right) for each cell type and patient. Eight different cell types are represented: MONO (green), PC (light blue), MBC (brown), CD8TC2 (blue), CD8TC1 (dark blue), CD8TC17 (black), CD8TREG (red) and CD4TC (orange). Three different patients are shown: SLE1 (asterisk), SLE2 (triangle), and SLE3 (circle) as indicated in the legend.

We applied SINGE and ONIDsc to the Mistry et al. (7) to compare the computational resources required by each method. We selected this dataset because it was small enough to allow both methods to be applied, while also being biologically more similar to our target dataset than those used in the benchmarking section. Eight different immune cell types were analyzed for each patient. Definitions of the cell types are provided in **Supplementary Table 1**. When examining total runtime as a function of the size of each of the 24 subsets, we found that SINGE (**Figures 4A, B**) took 18 times longer to run than ONIDsc (**Figures 4C, D**; **Table 1**), using the same computational resources (1 node, 8 CPUs, and 150 GB RAM). Moreover, our analysis showed an exponential increase in total runtime as dataset size increased linearly up to 9.8 MB for both methods (**Table 1**).

**Table 1:** Overview of methods applied to different datasets together with size in megabytes (MBs), gene and cell numbers, total run time in hours (hrs) and replica number (nr) of the biggest dataset analyzed.

Method	Lambdas	Dataset	Size (MBs)	Gene x cell (nr)	Total run time (hrs)	Replicas (nr)
SINGE	0, 0.01, 0.02, 0.05, 0.1	Deshpande and colleagues (30)	–	3025 x 3105	207.8	2
		Mistry and colleagues (7)	9.8	15873 x 1022	5077	8
ONIDsc	0.01	Nehar-Belaid and colleagues (11)	18.1	22550 x 1765	2421	
		Mistry and colleagues (7)	9.8	15873 x 1022	287	

To further characterize the algorithms, we explored additional factors that might explain the exponential increase in runtime. Specifically, we analyzed runtime as a function of the number of cells and genes per dataset independently for both methods. We found that gene count was more strongly correlated with total runtime than cell count for both SINGE (**Figure 4B**) and ONIDsc (**Figure 4D**). However, since all datasets in this study had more genes than cells, we cannot rule out a different trend in datasets where the cell count exceeds the gene count.

We also assessed SINGE’s scalability. When analyzing a 9.4 MB dataset with 15,400 genes using the Kay cluster (1 node, 8 CPUs), certain hyperparameter combinations, notably lambda = 0 combined with the smallest time resolution and number of lags, resulted in a runtime of 142 hours, which approached the maximum allowed by the longest-running cluster partition (LongQ; 144 hours). Increasing the number of CPUs to 40 on a single node reduced runtime only slightly (to 130 hours), and distributing 160 CPUs across 4 nodes did not improve runtime (**Table 2**). This indicates that using 1 node with 8 CPUs was the most resource-efficient configuration for SINGE, as it consumed the fewest core hours. It also highlights that SINGE is not scalable for datasets with more than 15,400 genes.

**Table 2:** Number of nodes, number of CPUs, run time (hours; hrs) and core hours (hrs) for a single hyperparameter combination (Lambda 0, time resolution 3, number of lags 5 and sigma 0.5) run with CD4TCs from a single SLE patient of the Mistry and colleagues (7) dataset using SINGE.

Number of nodes	Number of CPUs	Run time (hrs)	Core hours (hrs)
1	8	142	1136
1	40	130	5200
4	160	130	20800

Tests were performed on Kay cluster LongQ partition.

Altogether, this resource analysis showed that ONIDsc is significantly faster and more scalable than SINGE, primarily due to a reduced number of lambda hyperparameters tested and the exclusion of slower, suboptimal hyperparameter combinations.

#### Three SLE patient dataset: ONIDsc application

**Figure 5:**
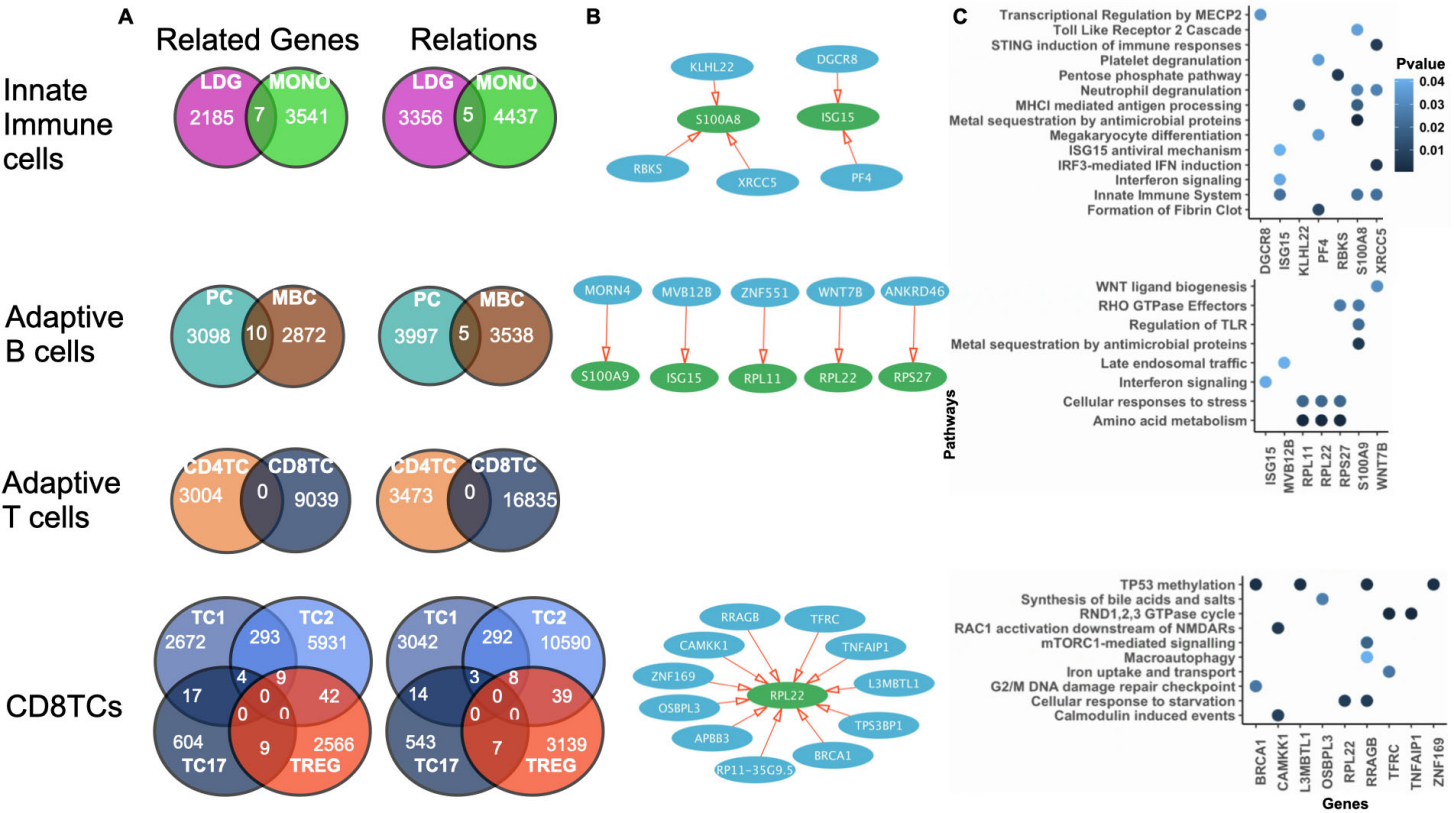
Shared regulatory networks and pathway enrichment across immune cell types in SLE patients. **(A)** Venn diagram of the number of related genes and relations common to innate immune cells, adaptive B cells, adaptive T cells, and CD8TCs for 2 out of the 3 patients analyzed in the Mistry et al. dataset. Cell types are colored as indicated in **Figure 4** caption. **(B)** Network of represented by a set of related genes (circles), which can be regulators (blue) or targets (green), and relations (red arrows). The gene name is written in white inside each circle (white). **(C)** PEA showing significant pathways of the related genes (P value< 0.05). P-value is indicated in blue. Common networks significantly involved in immune-related pathways for innate and adaptive immune cell types were identified.

**Figure 6:**
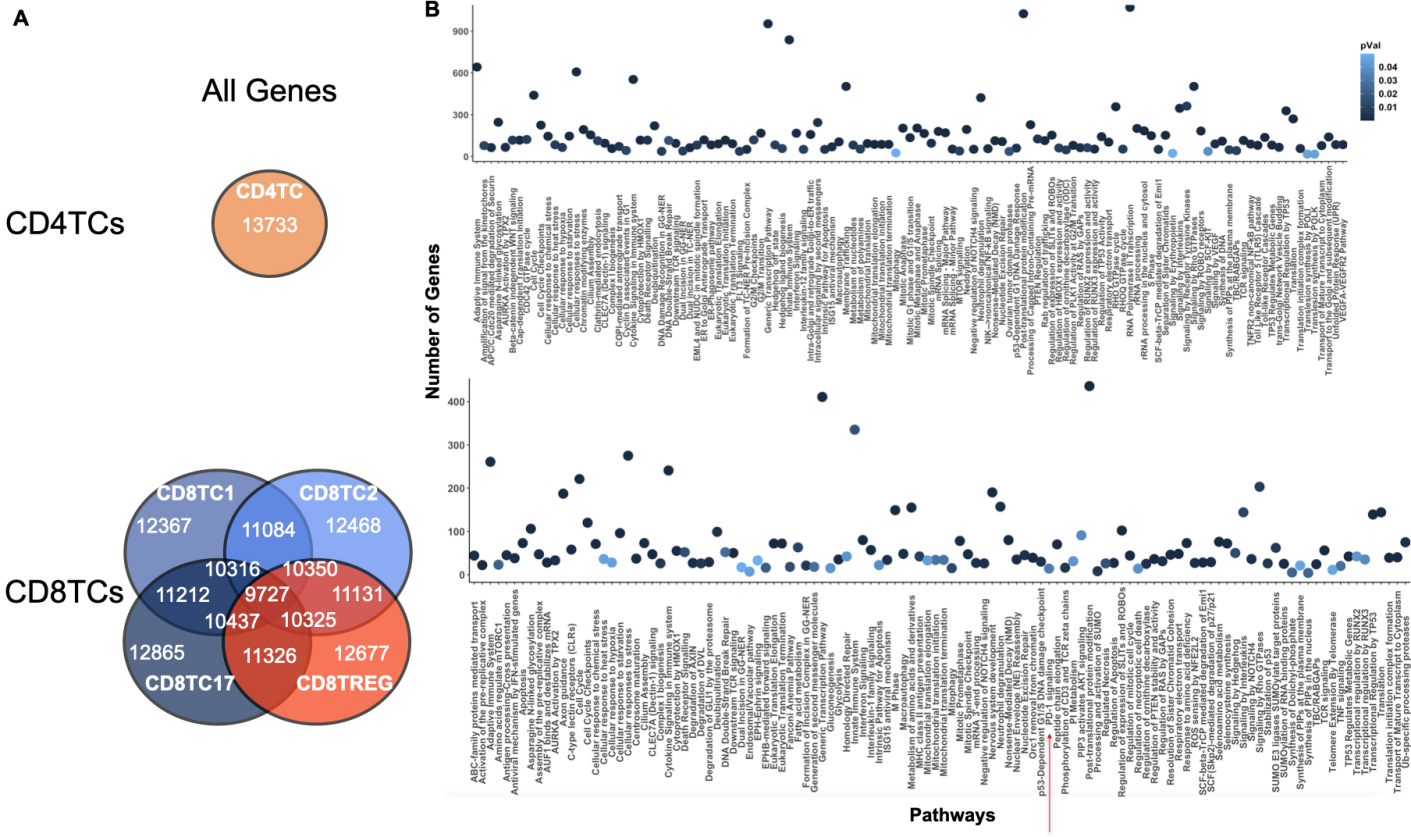
CD4TC and CD8TC gene overlap and pathway activation in SLE patients. **(A)** Venn diagram of the total number of genes (related and unrelated) common to CD4TCs and CD8TCs for two out of the three patients analyzed from the Mistry and colleagues dataset. Cell types are colored as indicated in **Figure 4** caption. **(B)** PEA showing significant pathways of all genes from intersecting CD8TCs (P value< 0.05). P-value is indicated in blue. CD8TC had signs of exhaustion, such as PD-1 signaling.

Analyzing the regulatory networks inferred using ONIDsc, we found networks shared by two out of the three SLE patients for each immune cell type analyzed. We then examined the intersecting related genes across major immune compartments: innate immune cells (MONOs and LDGs), adaptive B cells (PCs and MBCs), adaptive T cells (CD4TCs and CD8TCs), and adaptive CD8TC subtypes (**Figures 5A, B**). We studied these related genes using pathway enrichment analysis (PEA) and found evidence of innate immune cell activation, including neutrophil degranulation, platelet degranulation, metal sequestration by antimicrobial proteins, interferon (IFN) signaling, and regulation of toll-like receptors (TLRs). Similarly, in adaptive B lymphocytes, we observed metal sequestration by antimicrobial proteins, IFN signaling, and TLR regulation (**Figure 5C**). Importantly, these results are consistent with previous studies reporting overactivation of neutrophils, MONOs, and B lymphocytes in SLE (7, 63). We also found signs of CD8TC response to starvation and stress, including mTORC1-mediated signaling, macroautophagy, and activation of Ras-related C3 botulinum toxin substrate 1 (RAC1). Prior research has shown that autophagy is deregulated in SLE T lymphocytes (64), and that RAC protein dysregulation can impair T lymphocyte migration (65) and thymic development (66). We did not find networks shared across adaptive T lymphocytes, suggesting that CD4TCs and CD8TCs were dissimilar among the three patients. To gain further insight into the role of T lymphocytes, we analyzed CD4TCs and CD8TCs independently. First, we identified genes common to two out of the three SLE patients for each T cell type (**Figure 6A**). We then analyzed the intersection of CD4TC and CD8TC genes using PEA, and found evidence of activation, including VEGF signaling, p53 regulation, and T cell receptor (TCR) and cytokine signaling (**Figure 6B**). We also observed signs of CD4TC and CD8TC response to starvation and stress, such as macroautophagy-related pathways, and evidence of CD8TC exhaustion, including programmed cell death protein 1 (PD-1) signaling. This contrasts with the control dataset, where no signs of CD4TC or CD8TC stress or CD8TC exhaustion were observed. Therefore, these results are consistent with previous observations that CD4TCs are overactivated, CD8TCs are exhausted, and autophagy is deregulated in SLE T lymphocytes (13, 63). They also demonstrate the suitability and utility of ONIDsc for analyzing complex patient-derived single-cell datasets (14, 64).

#### Fifty-six SLE patient dataset: ONIDsc scalability

Next, we applied ONIDsc to the dataset from Nehar-Belaid and colleagues (10) to analyze the computational resources required by ONIDsc on the largest dataset identified to date in terms of both patient and gene numbers. Seven immune-related cell types were analyzed for each of the fifty-six SLE patients, resulting in a total of 392 subsets. We found between 2 and 1,889 cells per cell type, and between 2,997 and 22,577 genes (**Supplementary Figure 6A**). For comparison, Deshpande and colleagues (30), applied SINGE to a maximum of 3,105 genes, and a recent benchmarking study of eleven bulk-sequencing regulatory network inference algorithms analyzed up to 8,000 genes (34). In our case, the largest subset (18 MB) required 2,421 hours to run (**Table 1**; **Supplementary Figure 6B**).

**Figure 7:**
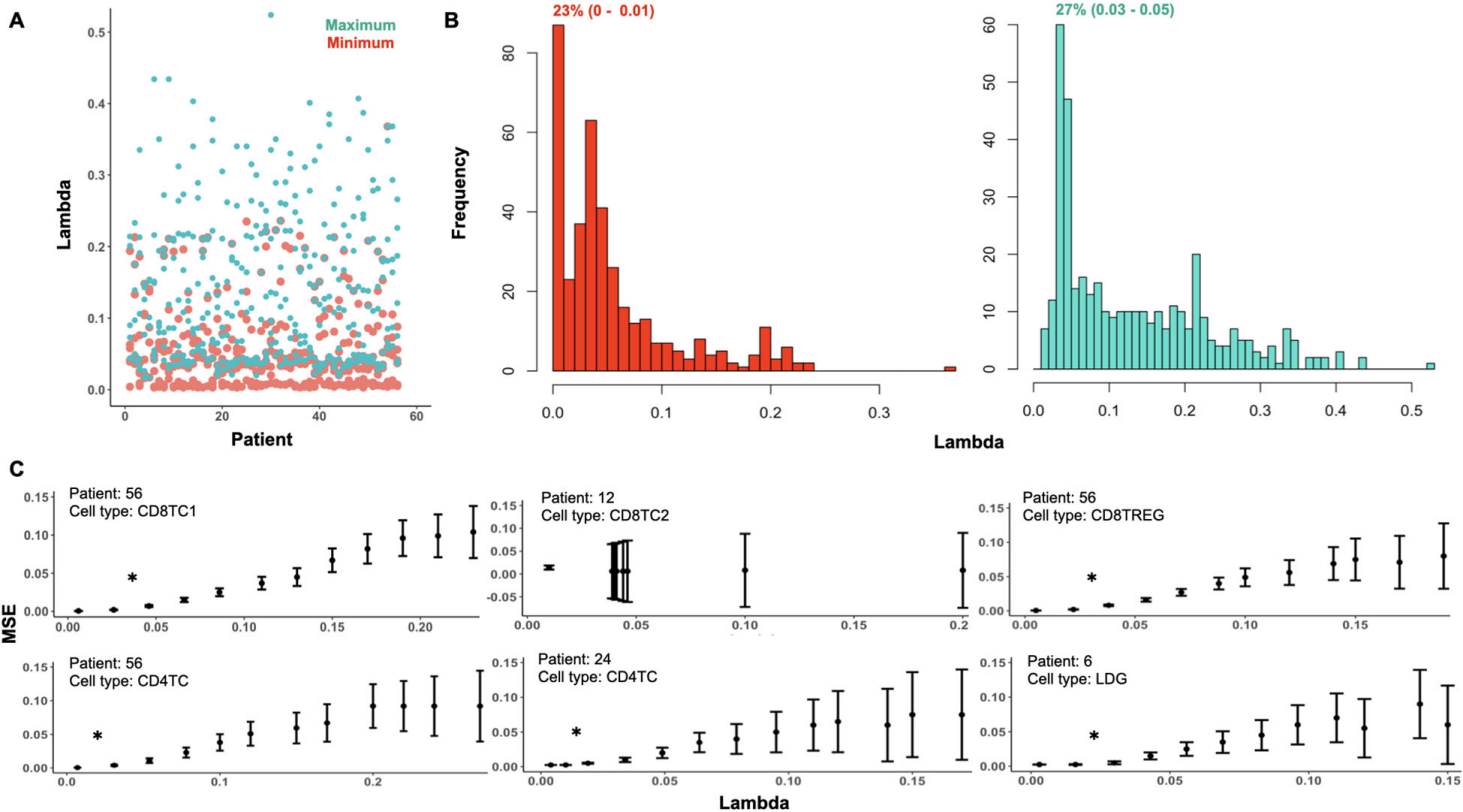
Optimal lambda distribution and MSE profiles across subsets in the 56-patient SLE dataset. **(A)** Minimum (red) and maximum (green) optimal lambda values per patient and **(B)** distribution of minimum and maximum optimal lambda values for all subsets analyzed from the Nehar-Belaid et all dataset. The percentage of subsets with the most frequent minimum and maximum lambda ranges are represented in red (23% (0 - 0.01)) and green (27% (0.03 - 0.05)), respectively. **(C)** MSE distribution over the range of optimal lambdas for six randomly sampled subsets: patient 56 CD8TC1, patient 56 CD4TC, patient 56 CD8TREG, patient 12 CD8TC2, patient 24 CD4TC and patient 6 LDG. The lambda distributions of these subsets are shown in **Supplementary Figure 8**. The interquartile range of MSE for each of the genes in the subset is represented by bars. A significant difference between two data points is indicated with an asterisk (P value< 0.05).

To apply ONIDsc, we first explored the topology of four patient subsets to assess their complexity. t-SNE plots revealed circular, simple single-path topologies (**Supplementary Figures 7A–D**). We then searched for the optimal lambda range across all datasets, aiming to identify a common optimal value that would enable patient-to-patient comparisons in downstream analyses. We found that the minimum and maximum lambda values followed a severely left-skewed distribution (**Figure 7A**). The most frequent minimum lambda ranged between 0 and 0.01 (23% of subsets), while the most frequent maximum lambda ranged between 0.03 and 0.05 (27% of subsets). We then examined MSE per gene across lambda values within the optimal range for six patient datasets (**Figure 7B**). For five of the six subsets, patient 56 CD8TC1, patient 56 CD4TC, patient 56 CD8TREG, patient 24 CD4TC, and patient 6 LDG, the MSE was close to zero at the individual minimum lambda values and increased significantly for lambdas above 0.02 (**Figure 7C**). This suggested that, for these subsets, minimum lambdas were more effective than maximum values in reducing MSE. Notably, patient 12 CD8TC2 showed no change in MSE across all lambda values, both within and outside the optimal range (**Supplementary Figure 8**), indicating that smaller lambdas did not increase MSE in this case. Considering all simulations, we selected 0.01 as the common optimal lambda, as it was the most frequent and minimized MSE across subsets.

**Figure 8:**
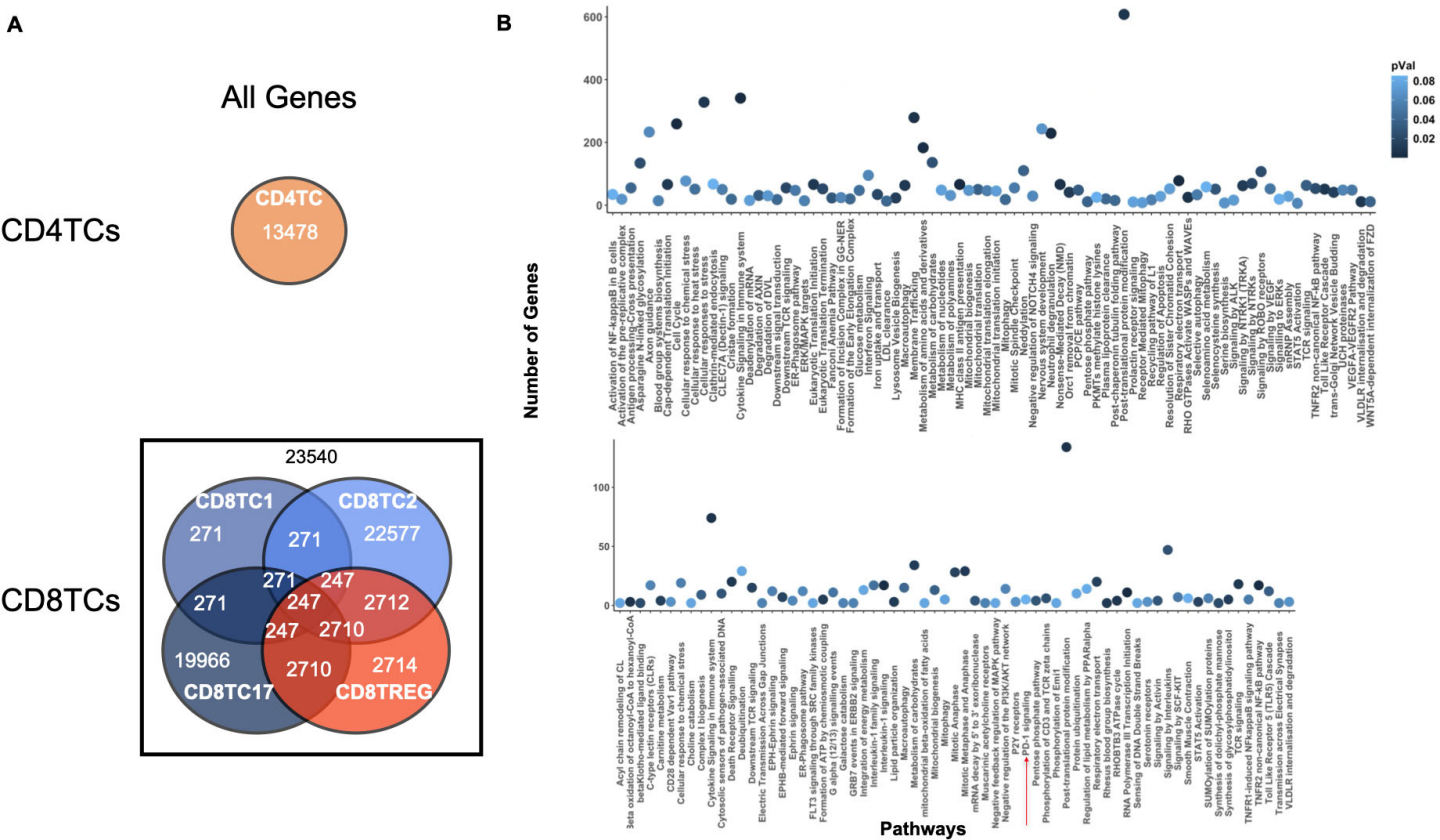
Common CD4TC and CD8TC genes and pathways across multiple SLE patients. **(A)** Venn diagram of the total number of genes (related and unrelated) common to CD4TCs and CD8TCs for two or more patients analyzed from the Nehar-Belaid and colleagues dataset. The number of genes present in all CD8TCs is shown in black. Cell types are colored as indicated in the **Figure 4** caption. **(B)** PEA showing significant pathways of all genes from intersecting CD8TCs (P value< 0.09). P-value is indicated in blue. CD8TCs had signs of exhaustion, such as PD-1 signaling.

To further test ONIDsc, we analyzed relations common to four out of the fifty-six patients for LDGs and MBCs (**Supplementary Figures 9**, **10A**). Using PEA, we found evidence of neutrophil activation (e.g., neutrophil degranulation, IFN signaling, and cytokine signaling) and MBC activation (e.g., TLR regulation) (**Supplementary Figures 9**, **10B**). These results were in line with previous observations that found neutrophils and B cells to be overactivated in SLE (7, 63). We also analyzed genes shared by at least two of the fifty-six patients for CD4TCs and CD8TCs (**Figure 8A**). The union of these genes was analyzed using PEA (**Figure 8B**). We found signs of CD4TC activation (e.g., antigen processing, TCR signaling, and cytokine signaling), CD8TC activation (e.g., antigen processing and TP53 regulation), CD8TC response to starvation and stress (e.g., macroautophagy), and CD8TC exhaustion (e.g., PD-1 signaling). These results again contrasted with the control dataset, where no signs of CD4TC or CD8TC stress or CD8TC exhaustion were observed, and were consistent with previous findings that CD4TCs are overactivated, CD8TCs are exhausted, and autophagy is deregulated in SLE T lymphocytes (14).

#### Fifty-six SLE patient dataset: ONIDsc predictions

**Figure 9:**
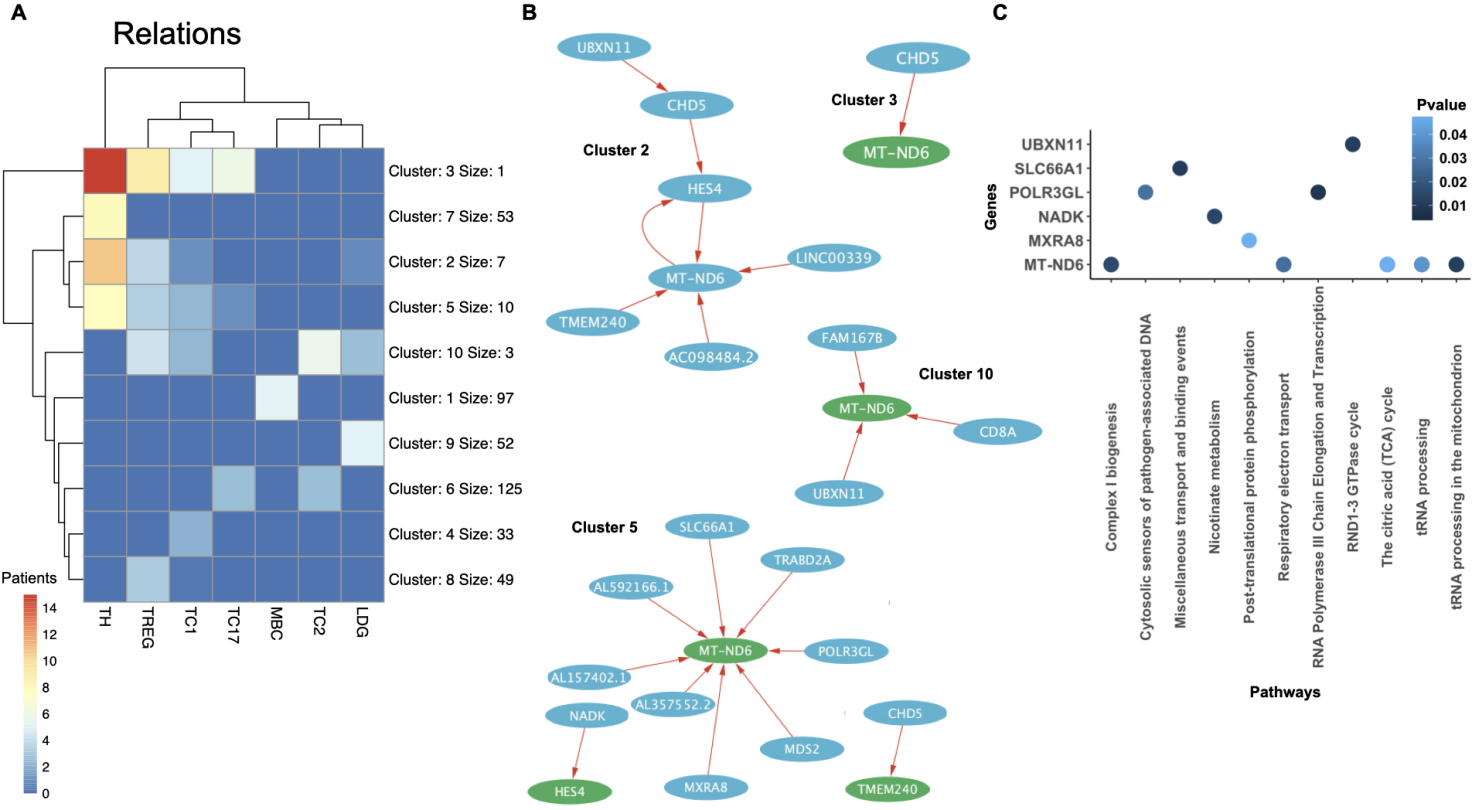
Clustering of shared regulatory relations across cell types and patients in SLE. **(A)** Clustering of the different cell types analyzed from the Nehar-Belaid and colleagues’ dataset. Each cluster is formed by a set of relations. The maximum number of patients with the same cluster is represented with colors that range from 0 (blue) to 14 (red). The most common genes per cell type were selected using a common threshold (**Table 3**). **(B)** Network of clusters 2, 3, 5 and 10 represented by a set of related genes (circles), which can be regulators (blue) or targets (green), and relations (red arrows). Regulators can affect one (light blue) or more (dark blue) targets. The name of the gene is written in white inside each circle (white). **(C)** PEA showing significant pathways of the network genes from clusters 2, 3, 5 and 10 (P value< 0.05). The p-value is indicated in blue.

Finally, we investigated shared gene regulatory relationships and disease-associated pathways across different SLE patients and immune cell types. We performed clustering of the cell types based on the most common regulatory relations among patients (**Figure 9A**). The unfiltered number of relations revealed a large and heterogeneous set of interactions between the different cell types (**Table 3**). We aimed to identify the most common relationships across both patients and cell types. To ensure a homogeneous number of nodes per cell type, we selected the top 60–105 most common genes per cell type, applying a variable common threshold across cell types (**Table 3**). The specific relations per cluster are provided in **Supplementary File 4**. Using a variable common threshold is a strategy commonly employed in systems with dynamic behavior, such as time series data analysis, where models may switch between regimes based on lagged variables (67) or in particle image processing, where it improves boundary detection, sizing accuracy, and shape parameter estimation (68). It must be noted that while this can lead to more nuanced, flexible and accurate decision-making, it can also increase complexity and potential biases. We found that most clusters per cell type exhibited low patient commonality, with the most common clusters (e.g., clusters 2, 3, 5, and 10) being present an average of seven patients (range: 3–15) across at least three of the five T cell types analyzed, a result expected given the high heterogeneity of SLE. We compared the most common SLE-specific relations (from clusters 2, 3, 5, and 10) with those found in control samples (from clusters 1, 3, and 4). Only one relation was shared between SLE and controls in CD4TCs: matrix remodeling-associated protein 8 (MXRA8) and MT-ND6. This suggests that the remaining common SLE relations are likely disease-specific and not part of normal immune function.

**Table 3:** Overview of most common networks per cell type.

Network features	TH	TREG	TC1	TC17	MBC	TC2	LDG
Unfiltered Nr of Relations	4310973	57583	1416	13207877	19933317	30454033	16285128
Common Variable Threshold	6	2	1	4	4	4	4
Genes	62	60	60	78	105	76	65
Relations	71	72	57	68	97	66	55

The unfiltered number of relations shows there is a large number of relations and a significant cell type heterogeneity.

We selected the most common relations across cell types ranging between 60-105. This was done using a common variable threshold defined as the minimum number of patients, out of the total 56 SLE patients analyzed, that had a given relation.

We performed PEA on all related nodes from the networks forming the most common clusters (**Figure 9B, C**). Six out of twenty-six genes were significantly associated with known pathways. MT-ND6, present in all clusters and cell types, was significantly linked to aerobic respiration pathways, including the TCA cycle and electron transport. Previous studies have shown that reduced MT-ND6 expression in SLE patients is associated with increased inflammatory CD4TC and CD8TREG death (69, 70). However, we also found MT-ND6 in all control cell types, suggesting its presence in SLE may reflect normal immune function rather than disease-specific activity. Solute carrier family 66 member 1 (SLC66A1), found in cluster 5 and present in CD4TC, CD8TC1, CD8TREG, and LDGs, is related to lysosomal transport. Encouragingly, previous studies have shown lysosomal transport dysfunction in phagocytes and B cells in SLE patients (71). Ultrabithorax Domain Protein 11 (UBXN11), found in clusters 2 and 10, was present in all cell types (**Supplementary Figures 9**–**15**) and is associated with the RND1–3 cycle in LDGs, CD8TREGs, and CD8TC1s. nicotinamide adenine dinucleotide kinase (NADK), RNA Polymerase III Subunit GL (POLR3GL), MXRA8, found in cluster 5, were present in CD4TC and CD8TREG and are linked to nicotinate metabolism, RNA transcription, and protein phosphorylation, respectively. Importantly, to the best of our knowledge, none of these four genes have been previously implicated in the pathophysiology of SLE. However, their functions are of potential relevance as they are involved in mechanisms deregulated in SLE. NADK is critical for redox balance in immune cells metabolism, POLR3GL may influence innate immune sensing of cytosolic DNA, a pathway known to be hyperactive in SLE, and MXRA8, a matrix remodeling protein, has been linked to viral entry and immune signaling (72). NADK, POLR3GL, and UBXN11 were not detected in the control datasets. Given their absence in controls and potential functional relevance, and the limited prior research on their roles in SLE, we propose MXRA8, NADK, POLR3GL, and UBXN11 as candidate genes for further investigation in the context of systemic lupus erythematosus.

## Discussion

We developed a regulatory network reconstruction algorithm, ONIDsc, that enhances GLG causality to identify molecular disease networks in SLE. This enhancement was achieved by applying cyclical coordinate descent to optimize the lambda penalty, improving model performance. GLG causality is based on SINGE, an algorithm well-suited for building networks from pseudotemporally ordered single-cell datasets with irregular time series. While SINGE includes subsampling, zero handling, and aggregation to improve robustness, it uses a limited set of predefined hyperparameter combinations, which can reduce performance, increase runtime, and limit scalability. Comparison with other network inference methods shows that ONIDsc consistently outperformed SINGE and, when assessing overall performance, ONIDsc also outperformed all the other network inference methods used to benchmark our algorithm for the ESC to endoderm differentiation dataset. This dataset’s gold standards were primarily derived from ChIP-chip and ChIP-seq data. Prior studies have shown that networks inferred using curated TF-gene interactions from ChIP-based experiments are more accurate than those based solely on gene expression data (73, 74). This may be because ChIP-derived interactions are direct (causal), while expression-based interactions can be indirect (correlational). In contrast, SINCERITIES, JUMP3, and GENIE3, which are optimized for gene expression data, outperformed ONIDsc on the retinoic acid dataset, where 48% of interactions came from LoF and GoF data.

ONIDsc is applicable across datasets when prior knowledge of cell type markers and process-driving genes is available. We used a supervised approach to improve accuracy and reliability, though this requires labeled data, which may be difficult to obtain. In such cases, unsupervised methods may be more appropriate, though they are harder to evaluate and more computationally intensive, especially for large datasets (75). We applied ONIDsc’s pseudotime inference to benchmarking datasets and found it performed significantly better than Monocle in early metrics when the dataset topology was linear or circular. However, pseudotime inference becomes more challenging in tree-like or bifurcated topologies (76, 77). While ONIDsc’s pseudotime method was suitable for the datasets studied, more complex topologies may require alternative approaches.

ONIDsc achieved higher precision, lower MSE, and faster runtimes than SINGE by selecting optimal and efficient hyperparameter combinations. A key advantage is its ability to scale up for larger patient datasets, which was not feasible with SINGE. Resource analysis showed that runtime correlated more strongly with gene count than cell count for both methods. However, since all subsets had more genes than cells, we cannot exclude different trends in datasets with more cells than genes. McCalla et al. (34) studied the effect of gene numbers (10 to 8000 genes) over run time for various network inference algorithms and similarly found an exponential increase. However, all datasets in their study had the same number of cells (5520). Therefore, the effect of different cell numbers on algorithm run time was not assessed. Interestingly, for datasets with more cells than genes, the exponential increase in run time when increasing genes linearly was conserved.

Applying ONIDsc to two SLE datasets demonstrated its ability to accurately reconstruct regulatory networks at scale (>15,000 genes)—a task not possible with SINGE. In both datasets, ONIDsc identified genes and pathways related to innate and adaptive B cell activation, consistent with previous findings of neutrophil, MONO, and B cell overactivation in SLE (7, 63). These cell types are involved in autoantibody production, antigen presentation, and cytokine secretion, all of which are dysregulated in SLE. In particular, TLR regulation is critical for immune tolerance, and its disruption can lead to autoimmunity (78). We also found signs of CD4TC activation, which contributes to autoantibody production and inflammation and is linked to SLE pathogenesis (14). Our data shows that CD8TCs were activated and exhausted due to the presence of the PD-1 signaling pathway. PD-1 is an inhibitory receptor expressed in response to continuous TCR stimulation without co-stimulatory molecules. In SLE, elevated PD-1 expression may impair CD8TC cytotoxicity, increasing infection risk and potentially triggering autoimmunity (13, 14). Finally, PD-1 is essential in maintaining CD8TC immune tolerance to tissue antigens by inhibiting T cell effector differentiation (79). Aerobic respiration pathways were enriched in CD8TCs and MBCs due to MT-ND6 expression. Reduced MT-ND6 levels in SLE have been linked to mitochondrial dysfunction, increased ROS, and ATP deficiency, promoting inflammatory T cell death (69, 70). Interestingly, we also found cell death pathways in CD8TREGs (**Supplementary Figure 12B**), suggesting that aerobic respiration may be relevant across all CD8TCs and MBCs. Moreover, the lysosomal transport pathway was associated with LDGs, CD4TCs, and most CD8TC subsets due to the presence of SLC66A1. Studies have shown this pathway to be dysfunctional in phagocytes and B cells in SLE patients (71, 80). Our findings suggest that lysosomal transport may also play a role in T cell dysregulation in SLE. The Rho GTPase signaling pathway, which regulates actin cytoskeleton dynamics and cell proliferation (81), was found to be significant in LDGs and some CD8TC subsets due to the presence of UBXN11. While RhoA GTPase has been proposed as a therapeutic target in SLE (82), UBXN11 has not previously been linked to the disease.

ONIDsc identified four genes, NADK, POLR3GL, MXRA8 and UBXN11, present in CD4TCs, CD8TREGs, CD8TC1s, and LDGs in SLE patients but absent in controls. The selected clusters containing these genes were shared by an average of seven patients, which is consistent with the high heterogeneity of SLE. Remarkably, these genes have not been shown before to be involved in SLE, but there is evidence that supports a role in the disease. These findings have also improved the robustness and generalizability of our method findings. Thus, these genes were associated with the nicotinate metabolism, RNA transcription, protein phosphorylation, and Rho GTPase signaling, respectively. A study of the literature strongly supports the role of these proteins in SLE, as they are involved in physiological functions commonly deregulated in this disease. The nicotinate metabolism pathway has been implicated in SLE and other inflammatory diseases (83). Interestingly, Serum metabolomic studies have shown that abnormalities in related pathways, such as nucleoside metabolism, may reflect immune cell dysfunction, particularly in energy-demanding processes like activation and proliferation (23). MXRA8, a cell surface adhesion receptor, is involved in extracellular matrix remodeling, endothelial and epithelial interactions, and viral entry (84). Dysregulation of MXRA8 may increase vascular permeability and immune cell infiltration, contributing to tissue damage in SLE (85). Finally, UBXN11, expressed in multiple immune cell types, belongs to a protein family known to regulate NF-κB and type I IFN pathways, both of which are central to SLE pathogenesis (86–88). Further experimental validation is needed to confirm the involvement of these genes in SLE. Nonetheless, our findings demonstrate that ONIDsc is a versatile and scalable tool for identifying physiological and pathological mechanisms in immune cells from complex single-cell datasets.

### Limitations of our study

Apart from the kernel sparsity, ONIDsc depends on three other hyperparameters that control the kernel smoothness and the window of past expression of candidate regulators. Currently, a reduced set of specific hyperparameter values and pairs are considered instead of all possible combinations. Thus, the algorithm could be further optimized by using grid search approaches to find optimal kernel hyperparameters. Nevertheless, it is not clear that this would yield significant performance improvements (30). Another way to improve ONIDsc algorithm could be by integrating different pseudotime-based methods. This could result in higher precision and certainty that the order really represents the process of study. ONIDsc current cell ordering method assumes linearity in the cell topology by ordering cells on a single dimension based on the normalized sum to the expression values of a set of predefined features, which are the main drivers of the process of study. Nevertheless, ONIDsc has the flexibility to incorporate different topologies and even combining real-time points with pseudotime inference methods. Other pseudotime algorithms, like tools for single cell analysis (TSCAN) (89), are better suited for linear topologies, while Monocle (28) or cell lineage and pseudotime inference for single-cell transcriptomics (Slingshot) (90) are better suited for tree-like topologies and selective locally linear inference of cellular expression relationships (SLICER) (91) is better suited for circular trajectories. Furthermore, cell trajectory inference using time-series single-cell RNA sequencing data (Tempora), has been developed to combine real-time points with pseudotime (92). Due to the different assumptions of the algorithm’s commonalities are not to be expected. Thus, the adequacy of the different cell ordering methods could be evaluated through benchmarking (93). Data exploration methods could be developed to assess which method is better suited for on a given dataset.

## Data Availability

The original contributions presented in the study are included in the article/**Supplementary Material**. Further inquiries can be directed to the corresponding author.
