# Supplementary material for: Optimized network inference for immune diseased single cells
Source: Front Immunol. 2025 Jul 24;16:1597862. doi: 10.3389/fimmu.2025.1597862 (PMC12328306; doi:10.3389/fimmu.2025.1597862)
Supplement: Supplementary file 1 [file DataSheet1.pdf]

Supplementary Information:

Supplementary Material 1: Description of preprocessing and parameter setting used for all patient datasets FAST-Q files of short-read Illumina sequences were aligned to a human reference genome (GrCh38) using STAR. Cell Ranger output files were loaded into R for analysis using Seurat **(1)**. Gene and cell numbers, UMI counts, and percentage of mitochondrial gene expression were used for quality control and filtering as follows. Genes were filtered based on expression by a minimum number of cells (3), and cells were filtered based on a minimum number of genes (100). Doublets or multiplets were filtered by a maximum number of UMI counts (1000), and empty droplets were also filtered by a minimum number of UMI counts (200). Finally, low-quality or dead cells were removed based on the maximum percentage of mitochondrial gene expression **(5)**.

No imputation was performed, since inappropriate imputation can negatively impact pseudotime inference depending on the choice of algorithm **(2)**. Furthermore, the GLG algorithm does not require prior imputation of missing values due to its kernel-based approach for analysing irregular time series. No Normalization was performed as this was a part of ONIDsc Downstream Analysis Pipeline.

Supplementary Files:

Supplementary File 1: Run time and core optimization analysis of the Mistry and colleagues dataset using SINGE algorithm is in GSE142016 CoreOptimization SINGE.xlsx

Supplementary File 2: Run time and core optimization analysis of the Mistry and colleagues' dataset using the ONIDsc algorithm is in GSE142016 CoreOptimization_ONIDsc.xlsx.

Supplementary File 3: Run time and core optimization analysis of the Nehar-Belaid and colleagues dataset using ONIDsc algorithm is in Pascual_CoreOptimization_ONIDsc.xlsx.

Supplementary File 4: Clustered relation analysis of the Nehar-Belaid and colleagues' dataset is in ONIDsc_Pascual_clusters.csv.

Supplementary Figures and Tables:

Cell Type Markers

Supplementary Table 1: Description of cell markers used to define the different immune cell types.

**Cell types**	**Cell markers**
LDG	Fc gamma receptor 3B (FCGR3B)
CD8TREG	Cluster of differentiation (CD) 8, (interleukin 2 receptor alpha) IL-2Ra, cytotoxic T-lymphocyte-associated protein (CTLA) 4, forkhead box protein (FOXP) 3, IL10
CD8TC17	CD8, C-C motif chemokine receptor (CCR) 6, killer cell lectin-like receptor subfamily B member (KLRB) 1, IL-17, Interferon Regulatory Factor (IRF) 4, retinoic acid receptor-related orphan receptor C (RORC)
CD8TC2	CD8, IL-4, IL-4R, IL-4I1, IL-5, CCR4, globin transcription factor (GATA) 3
CD8TC1	CD8, tumor necrosis factor alpha (TNFa), IL-2, CXCR3
CD4TC	CD4
MBC	CD20, CD40, CD80, PDL-2, CXCR3-6, CD19, CD25, CD30
PC	CD27, CD38, CD78, CD138, CD319, IL-6
MONO	CD14, CD16

Lasso optimization

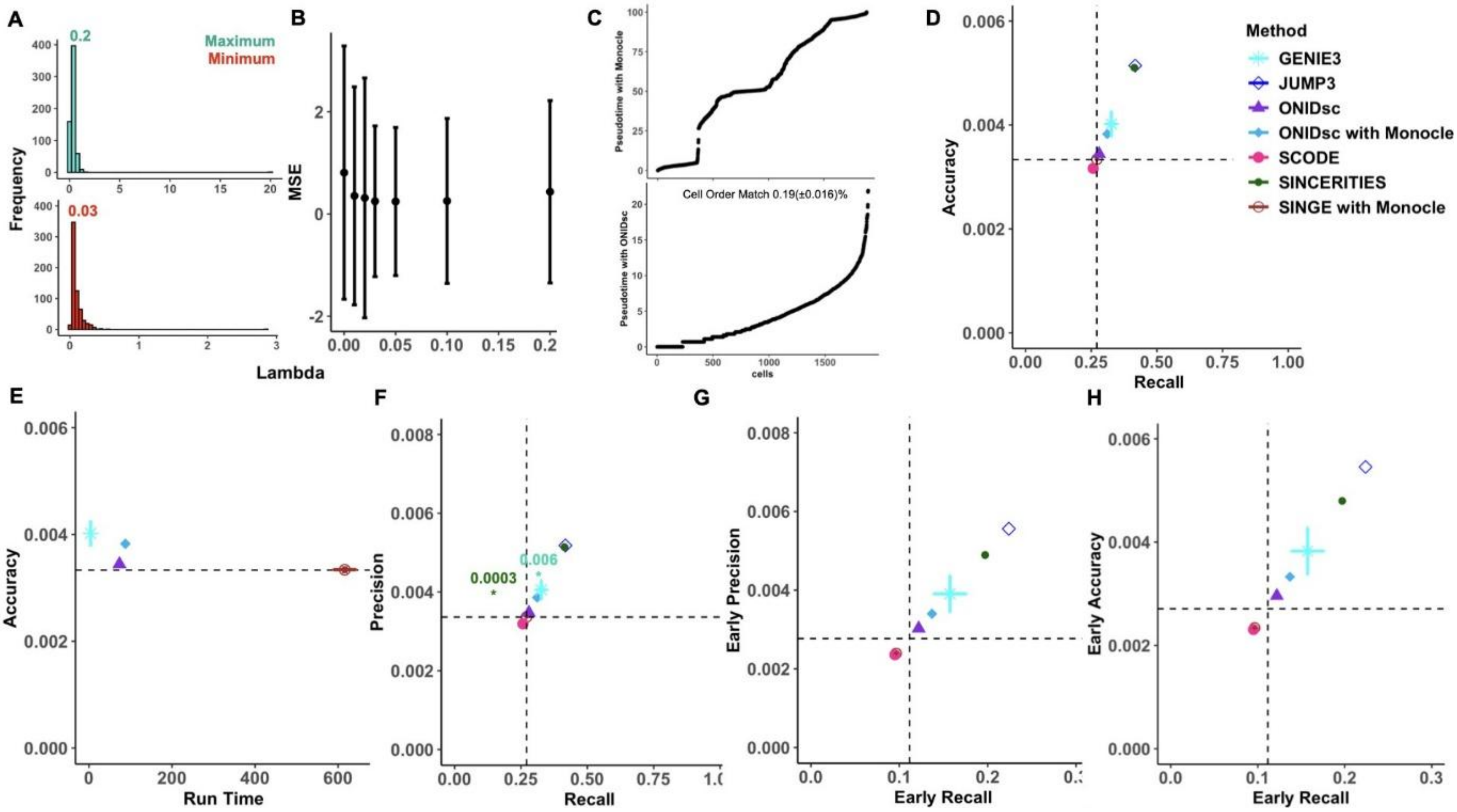

Supplementary Figure 1: Benchmarking of ONIDsc pseudotemporal and network inference algorithms against five other network inference and a pseudotemporal algorithms on the retinoic acid dataset from Semrau and colleagues. **(A)** Minimum (red) and maximum (green) optimal lambdas. **(B)** MSE distribution over lambdas proposed by SINGE and ONIDsc. **(C)** Comparison of ONIDsc and Monocle pseudotime inference. Cell Order Match indicates the percentage of cells with similar order in both pseudotemporal methods when allowing a degree of variability of 0.016%, which accounts for up to 30 positions in each direction in the single-dimensional cell order vector with 1886 total number of positions. **(D)** Accuracy over recall, **(E)** accuracy over run time, **(F)** precision over recall, **(G)** early precision over early recall and **(H)** early accuracy over early recall. Six GRN Inference algorithms were tested: GENIE3 (light blue), JUMP3 (dark blue), ONIDsc full method (purple), ONIDsc's network inference combined with Monocle's pseudotime (blue), SCODE (fuchsia), SINCERITIES (dark green) and SINGE combined with Monocle's pseudotime (brown). Four replicas were run for GENIE3, ONIDsc and SINGE. The standard deviation is represented with bars. T-tests comparing the average precision of ONIDsc, with GENIE3 and SINGE were performed. Statistically significant differences (P value < 0.05) were represented with an asterisk, and the p value is indicated above. Both asterisks and p values are colored by the method ONIDsc was compared. Methods are also represented with different shapes as indicated in the

figure legend. ONIDsc pseudotime inference was the fifth method in all metrics and ONIDsc network inference was the fourth best method for the late metrics and the third best, tied with GENIE3 for the early metrics.

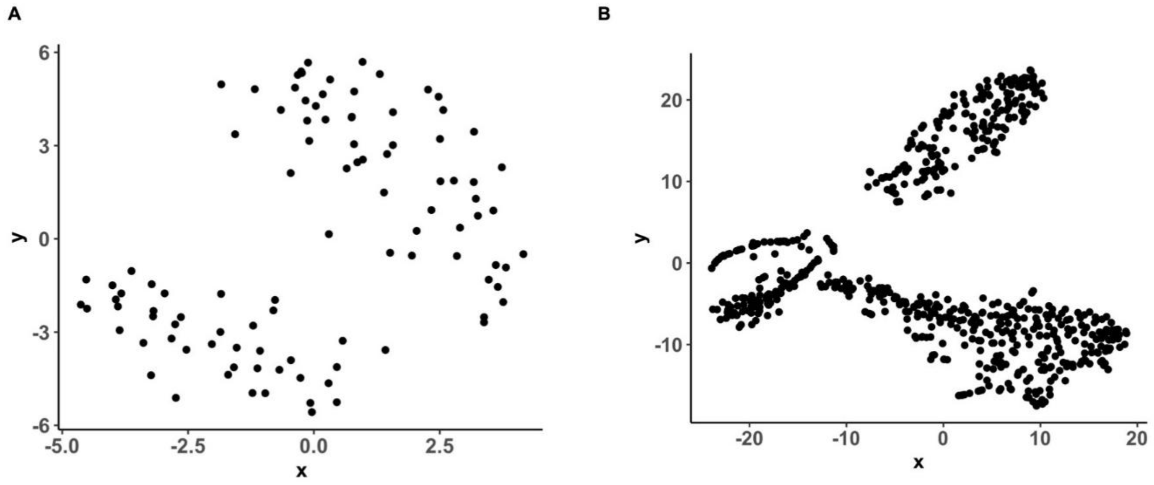

Supplementary Figure 2: Visualization of the benchmarking datasets' topology for **(A)** ESC-to-endoderm differentiation dataset from Hayashi and colleagues had a linear or single path topology, and **(B)** Retinoic acid dataset from Semrau and colleagues **(3)** had a tree-like, bifurcated or multiple path topology. t-SNE method was used to reduce non-linear high-dimensional datasets into two dimensions (x and y-axis).

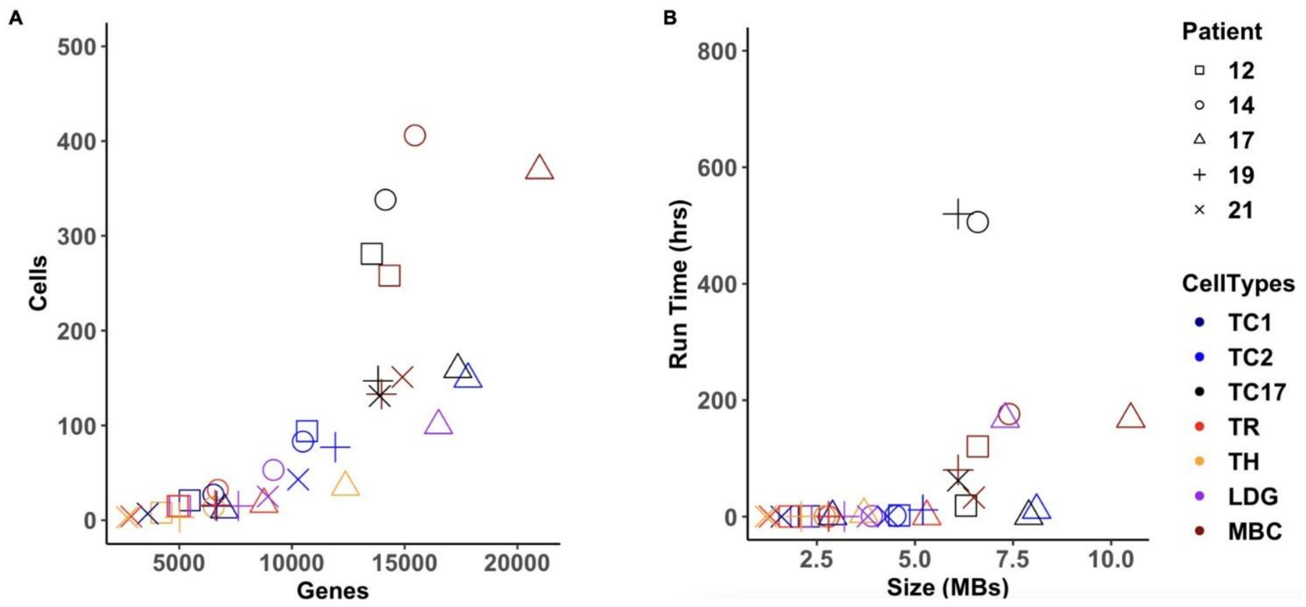

Supplementary Figure 3: (A) Cell as function of gene number for each cell type and patient analyzed from the Schafflick and colleagues' dataset. Seven cell types are represented: CD8TC1 (dark blue), CD8TC2 (blue), CD8TC17 (black), CD8TREG (red), CD4TC (orange), LDG (purple) and MBC (brown). Five IIH patients are represented using symbols and letters as indicated in the legend. **(B)** Total run time in hours (hrs) as a function of subset size in megabytes (MBs) per cell type and patient.

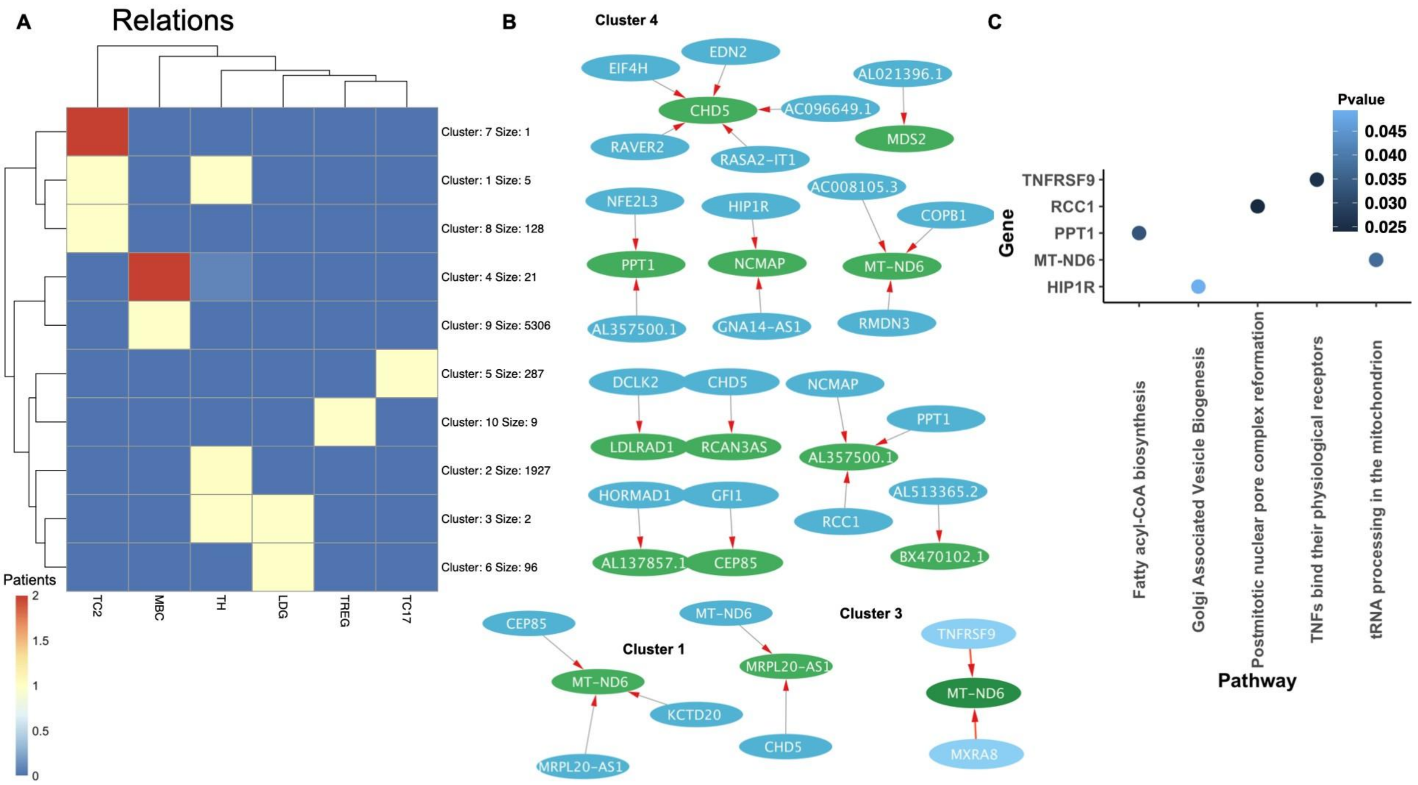

Supplementary Figure 4: (A) Clustering of the different cell types analyzed from the Schafflick and colleague's dataset. Each cluster is formed by a set of relations. The maximum number of patients with the same cluster is represented with colors that range from 0 (blue) to 2 (red). No minimum number of common patient threshold was applied. **(B)** Network of clusters 1, 3 and 4 represented by a set of related genes (circles), which can be regulators (blue) or targets (green), and relations (red arrows). Regulators can affect one (light blue) or more (dark blue) targets. The name of the gene is written in white inside each circle (white). **(C)** PEA showing significant pathways of the network genes from clusters 1, 3 and 4 (P value < 0.05). The p-value is indicated in blue**.**

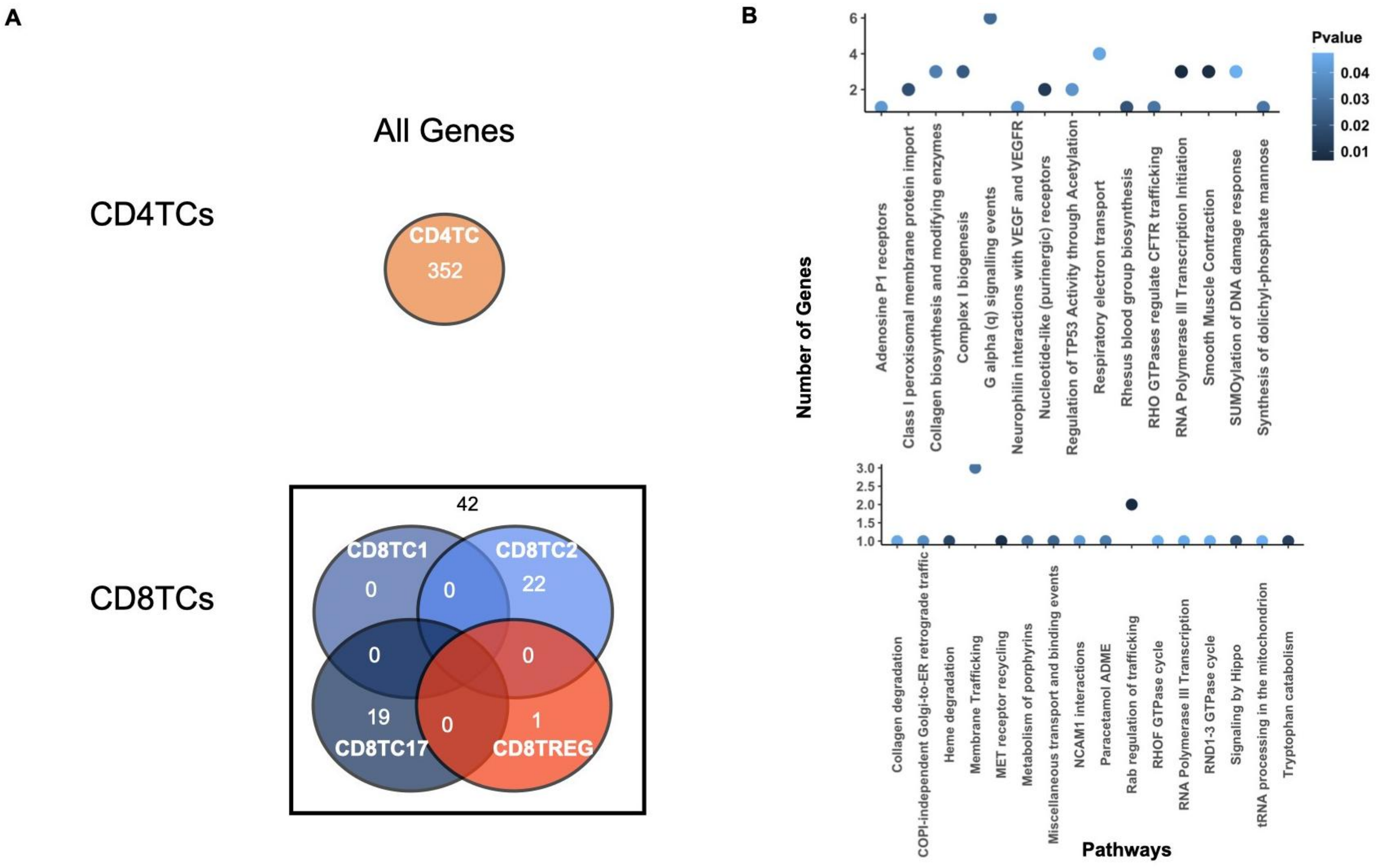

Supplementary Figure 5: (A) Venn diagram of the total number of genes present in CD4TCs and CD8TCs from one or more controls analyzed in the Schafflick and colleague's dataset. The number of genes present in all CD8TCs is shown in black. Cell types are colored as indicated in Figure 4 caption. **(B)** PEA showing significant pathways of all genes common to CD4TCs and CD8TCs found in one or more patients (P value < 0.05). P value is indicated in blue. We found no sign of CD8TC exhaustion.

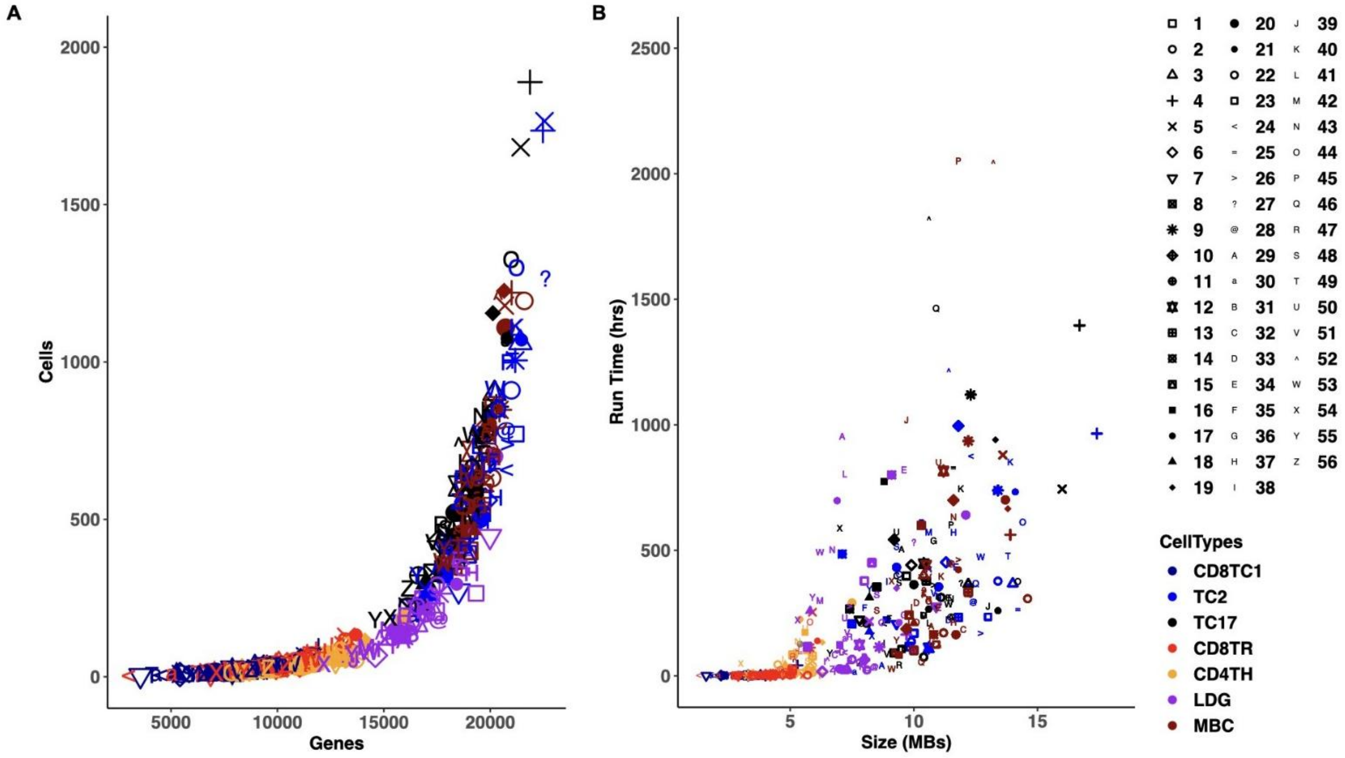

Supplementary Figure 6: (A) Cell as function of gene number for each cell type and patient analyzed from the Nehar-Belaid and colleagues' dataset (Table 3). Seven different cell types are represented: CD8TC1 (dark blue), CD8TC2 (blue), CD8TC17 (black), CD8TREG (red), CD4TC (orange), LDG (purple) and MBC (brown). Fifty-six different patients are represented using symbols and letters as indicated in the legend. **(B)** Total run time in hours (hrs) as a function of subset size in megabytes (MBs) for each cell type and patient.

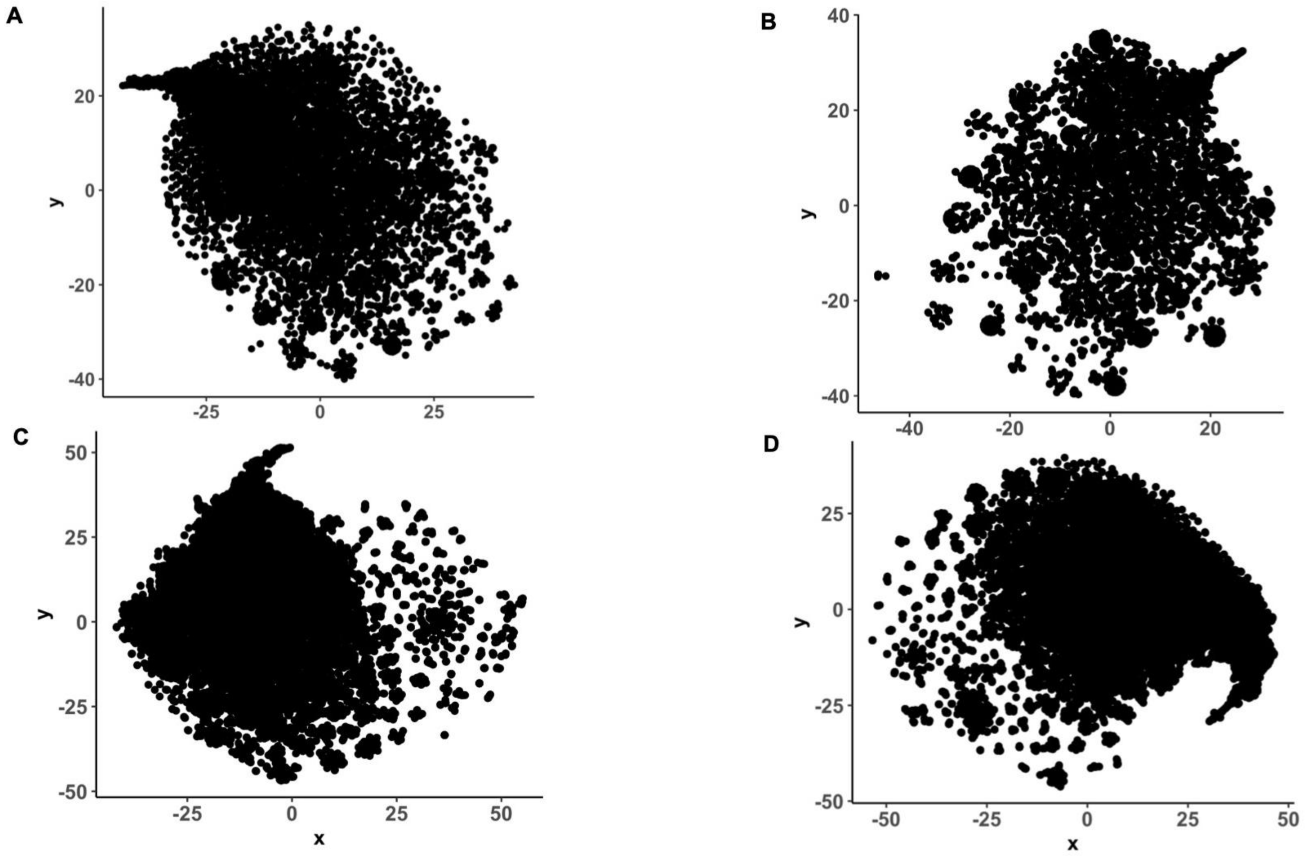

Supplementary Figure 7: Visualization of the topology of four data subsets from the Nehar-Belaid and colleagues' dataset. The different subsets displayed circular single path topology: **(A)** Patient 1 CD4TC, **(B)** Patient 3 CD8TC1, **(C)** Patient 3 LDG, **(D)** Patient 5 LDG. t-SNE method was used to reduce non-linear high dimensional datasets into two dimensions (x and y-axis).

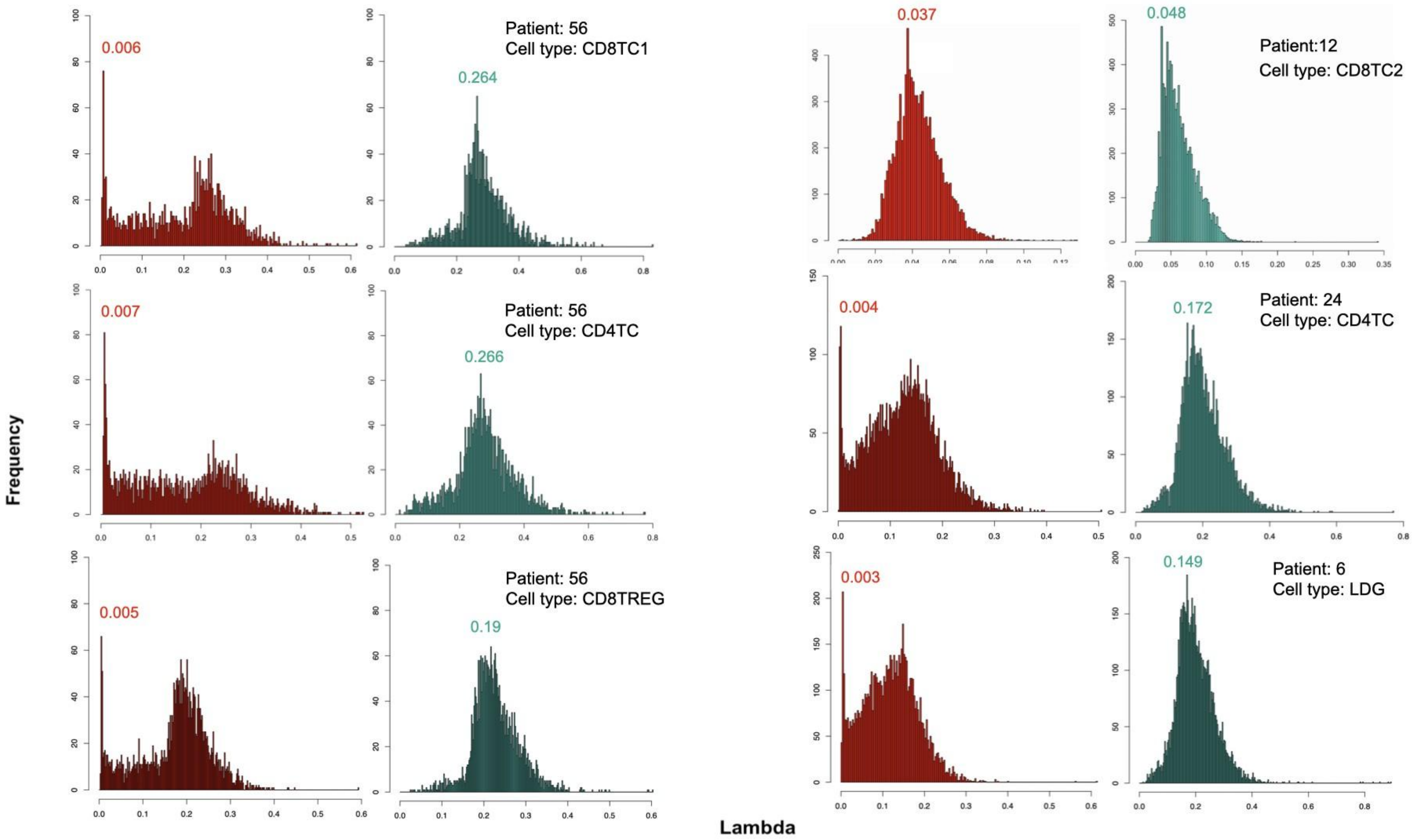

Supplementary Figure 8: Minimum (red) and maximum (green) lambda distribution for six different subsets for which the MSE was also analyzed (see Figure 7B). The most frequent minimum and maximum lambda values are typed in red and green, respectively.

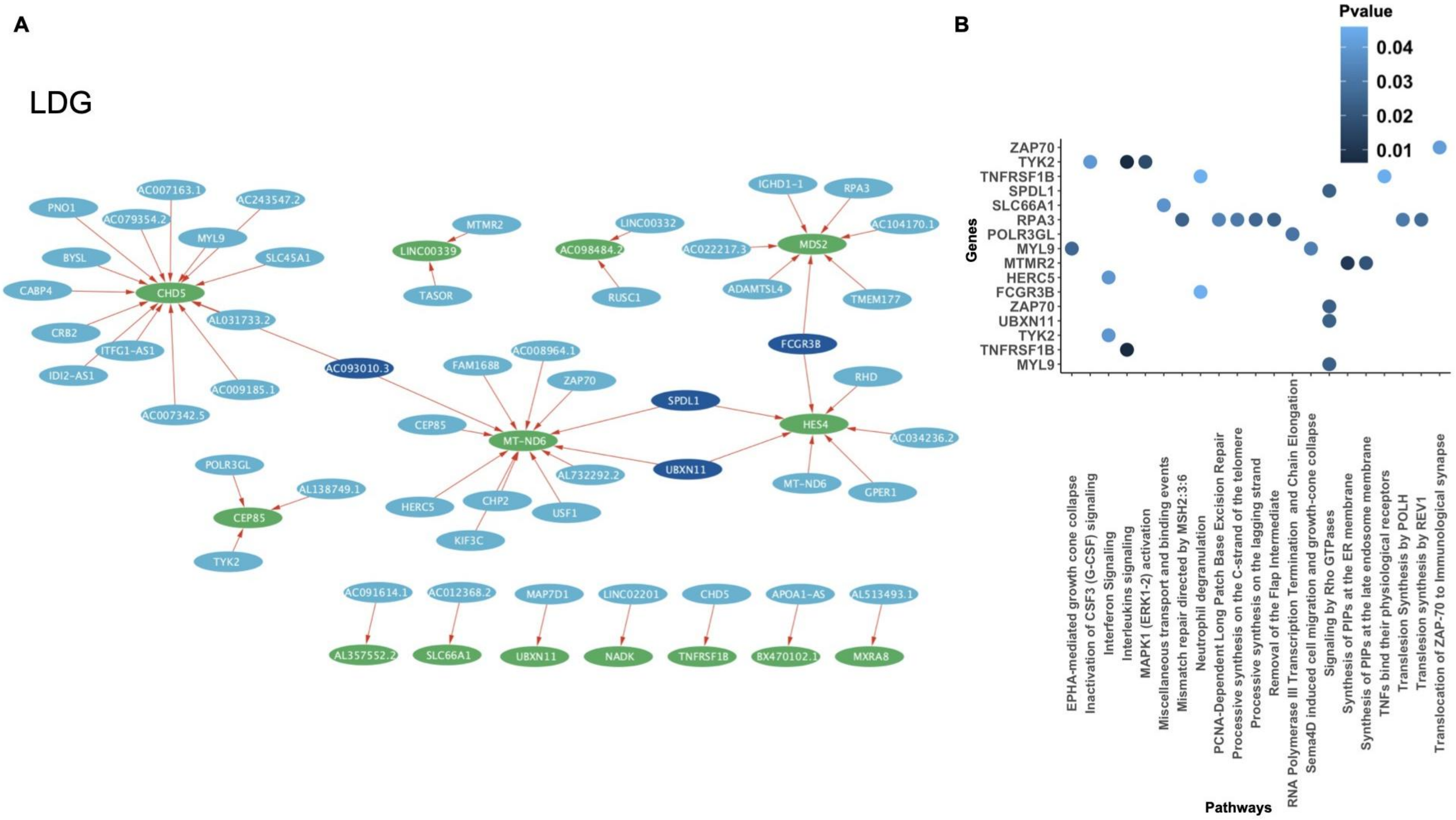

Supplementary Figure 9: (A) Network and **(B)** significant (P value < 0.05) genes and pathways after PEA of LDG cell type analyzed from the Nehar-Belaid and colleagues' dataset. The network is represented by a set of genes (circles), which can be regulators (blue) or targets (green), and relations (red arrows). Regulators can affect one (light blue) or more (dark blue) targets. The name of the gene is written in white inside each circle (white). The p-value is indicated in blue.

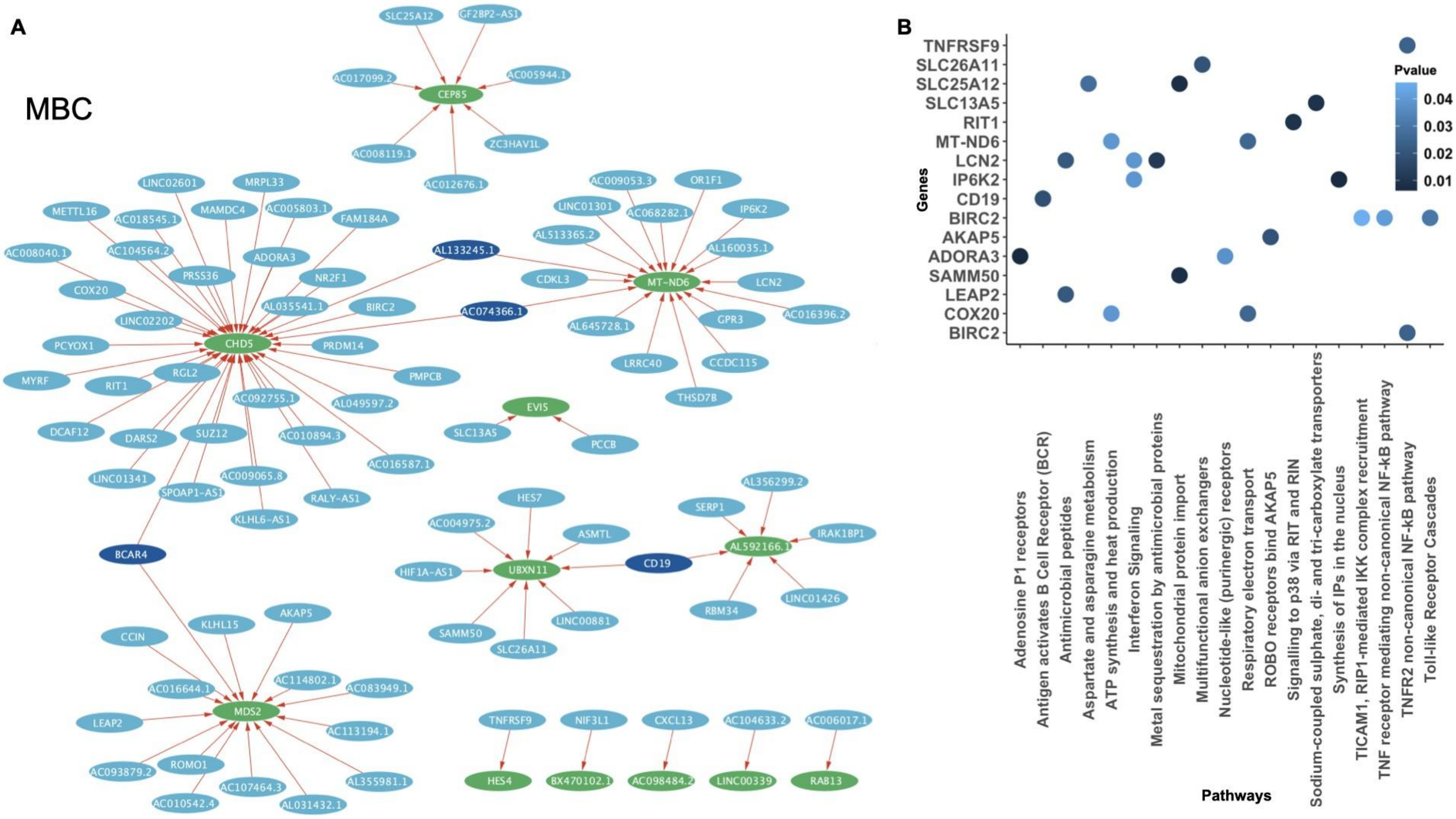

Supplementary Figure 10: (A) Network and **(B)** significant (P value < 0.05) genes and pathways after PEA of MBC cell type analyzed from the Nehar-Belaid and colleagues' dataset. For further description of the network colors see Supplementary Figure 9 caption. The p-value is indicated in blue.

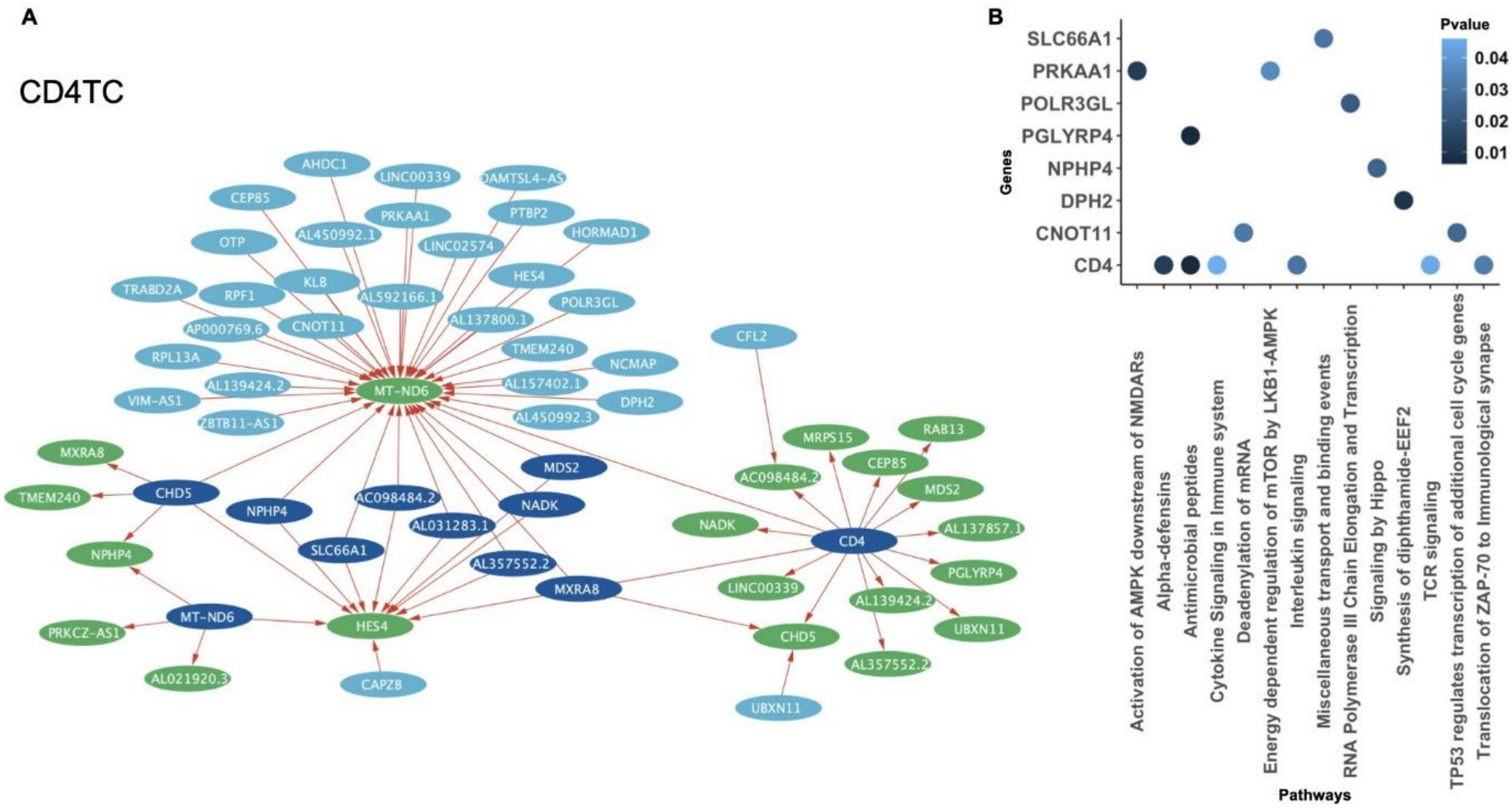

Supplementary Figure 11: (A) Network and **(B)** significant (P value < 0.05) genes and pathways after PEA of CD4TC cell type analyzed from the Nehar-Belaid and colleagues' dataset. For further description of the network colors see Supplementary Figure 9 caption. The p-value is indicated in blue.

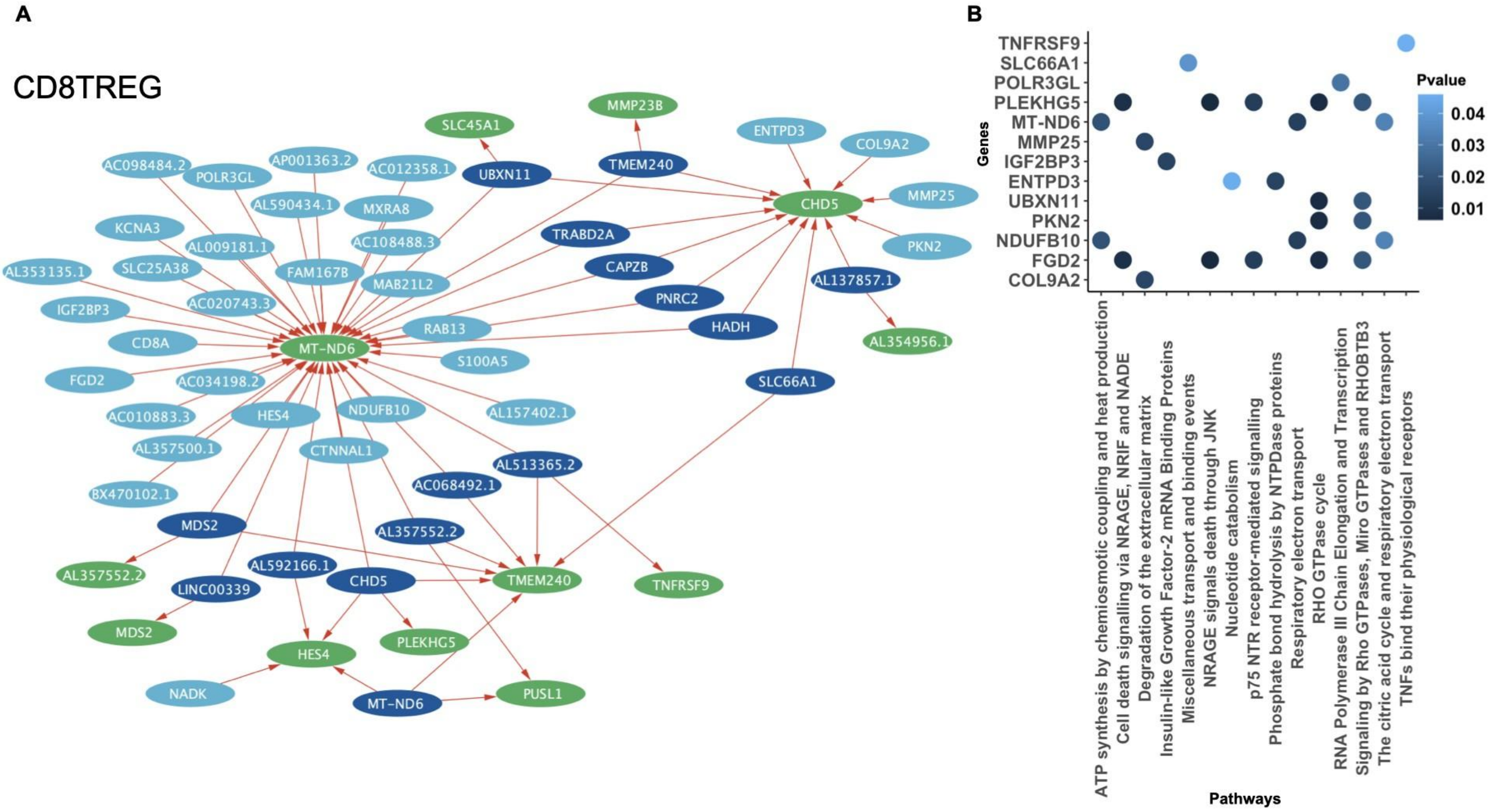

Supplementary Figure 12: (A) Network and **(B)** significant (P value < 0.05) genes and pathways after PEA of CD8TREG cell type analyzed from the Nehar-Belaid and colleagues' dataset. For further description of the network colors see Supplementary Figure 9 caption. The p-value is indicated in blue.

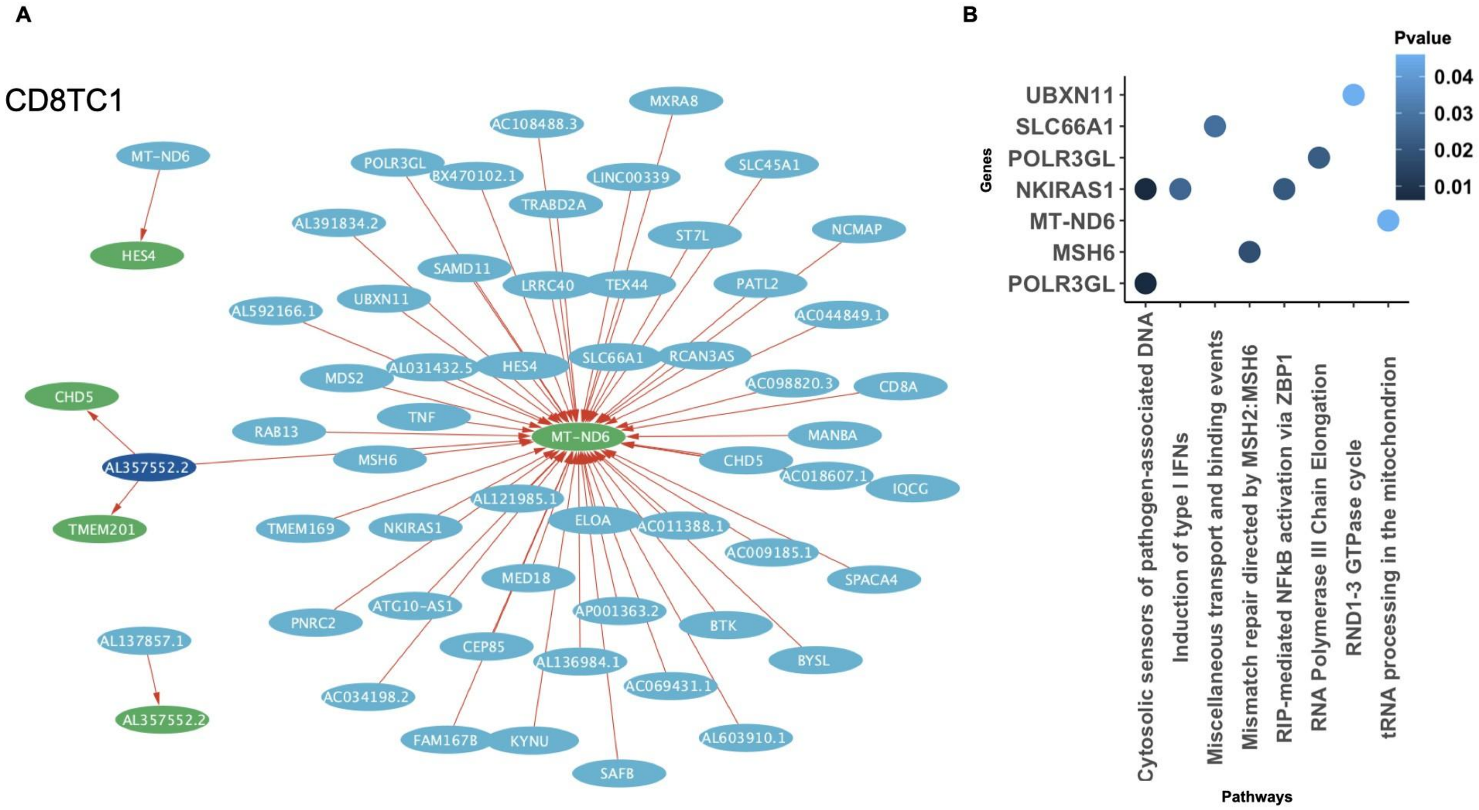

Supplementary Figure 13: (A) Network and **(B)** significant (P value < 0.05) genes and pathways after PEA of CD8TC1 cell type analyzed from the Nehar-Belaid and colleagues' dataset. For further description of the network colors see Supplementary Figure 9 caption. The p-value is indicated in blue.

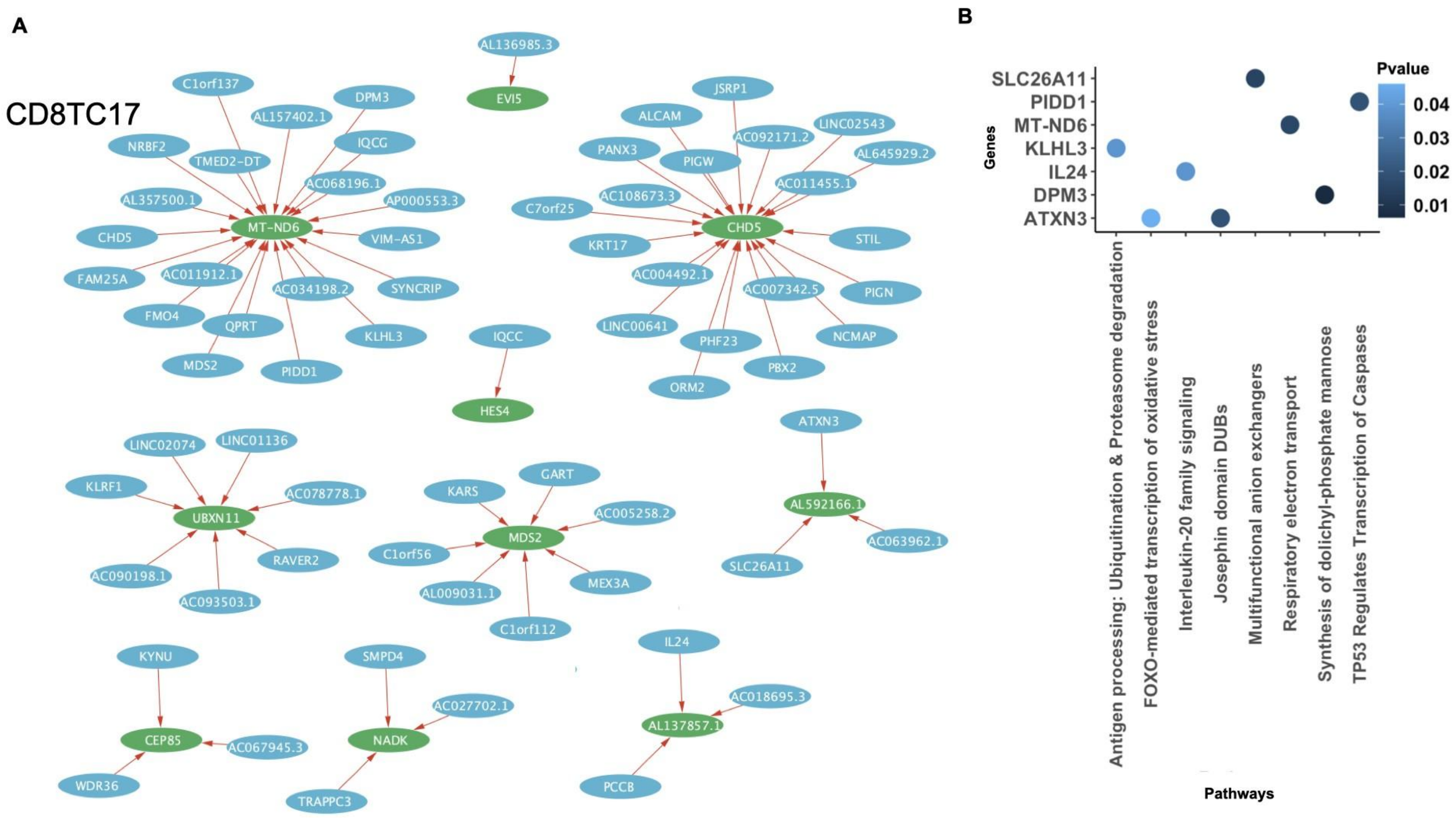

Supplementary Figure 14: (A) Network and **(B)** significant (P value < 0.05) genes and pathways after PEA of CD8TC17 cell type analyzed from the Nehar-Belaid and colleagues' dataset. For further description of the network colors see Supplementary Figure 9 caption. The p-value is indicated in blue.

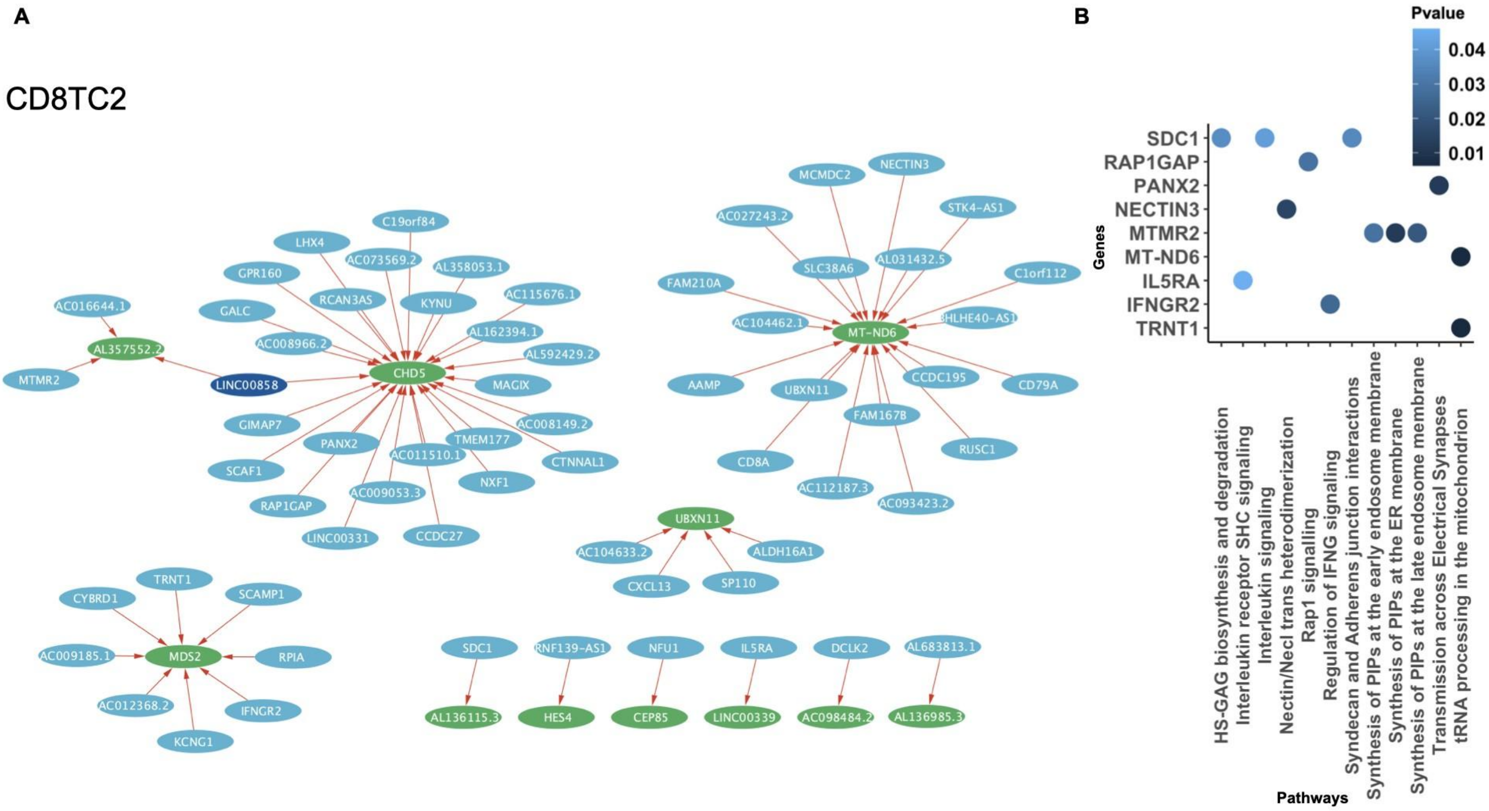

Supplementary Figure 15: (A) Network and **(B)** significant (P value < 0.05) genes and pathways after PEA of CD8TC2 cell type analyzed from the Nehar-Belaid and colleagues' dataset. For further description of the network colors see Supplementary Figure 9 caption. The p-value is indicated in blue.

References

1. Butler A, Hoffman P, Smibert P, Papalexi E, Satija R. Integrating single-cell transcriptomic data across different conditions, technologies, and species. Nature. 2018;36(5):411-23. doi:10.1038/nbt.4096.
2. Zhang J, Nie Q, Zhou T. Topographer reveals dynamic mechanisms of cell fate decisions from single-cell transcriptomic data. bioRxive. 2018. doi:10.1101/251207.
3. Semrau S, Goldmann JE, Soumillon M, Mikkelsen TS, Jaenisch R, van Oudenaarden A. Dynamics of lineage commitment revealed by single-cell transcriptomics of differentiating embryonic stem cells. Nature communications. 2017;8(1096). doi:10.1038/s41467-017-01076-4.
